# PPE51 modulates membrane integrity in *Mycobacterium marinum*

**DOI:** 10.1128/mbio.01044-25

**Published:** 2025-09-22

**Authors:** Vicky Charitou, Beatriz Izquierdo Lafuente, Eva Habjan, Coen Kuijl, Joost J. Willemse, Wilbert Bitter, Alexander Speer

**Affiliations:** 1 Department of Medical Microbiology and Infection Control, Amsterdam UMC, Location VU Medical Center, Amsterdam, the Netherlands; 2 Section of Molecular Microbiology, Amsterdam Institute of Molecular and Life Sciences (AIMMS), Vrije Universiteit Amsterdam, Amsterdam, the Netherlands; 3 Institute of Biology, Leiden University, Sylvius Laboratory, Leiden, the Netherlands; Max Planck Institute for Infection Biology, Berlin, Germany

**Keywords:** *Mycobacterium*, protein secretion system, type VII secretion system, *Mycobacterium marinum*, nutrient transport, outer membrane proteins, cell-wall integrity, PPE51

## Abstract

PPE proteins, which are exclusive to mycobacteria, are substrates of type VII secretion systems, also unique to this genus. Their function has been an enigma for a long time, but has recently received attention for their association with nutrient uptake across the mycobacterial outer membrane. One of these nutrient transporters is PPE51, involved in glycerol and glucose uptake in *Mycobacterium tuberculosis*. We investigated the role of PPE51 proteins in nutrient uptake and membrane integrity using the model organism *Mycobacterium marinum*, whose genome encodes four paralogs. We created single, double, and triple mutants of *ppe51* and noted that the presence of the final *ppe51* gene was essential. To determine if PPE51’s function is conserved in *M. marinum*, we investigated its role in glucose and glycerol uptake and found it to be retained across species. Further analysis of its secretion mechanism revealed that PPE51 proteins are ESX-5 substrates, one of the type VII secretion systems, in line with its proposed role in nutrient transport. Notably, and in contrast to its role in glycerol transport, the *ppe51* mutants presented substantially altered membrane permeability, as observed by ethidium bromide influx and increased susceptibility to high-molecular-weight antibiotics, such as rifampicin and vancomycin. Scanning electron microscopy showed that the triple mutant has gross alterations in cell morphology. Furthermore, the triple mutant demonstrated attenuation during infection experiments in murine macrophages, likely due to loss of cell-wall integrity. In summary, we analyzed the characteristics of PPE51 in more detail, revealing a new role in membrane integrity and an indirect link with virulence.

**IMPORTANCE:**

The impermeable outer membrane of pathogenic mycobacteria presents a major obstacle to nutrient acquisition and antibiotic penetration. PPE51, a substrate of the ESX-5 secretion system, has previously been linked to glucose and glycerol uptake. Our study in *Mycobacterium marinum* reveals an unexpected additional role for PPE51 in maintaining membrane integrity. Loss of PPE51 not only impairs nutrient uptake but also causes increased membrane permeability, altered antibiotic susceptibility, and reduced virulence. These findings redefine PPE51 as more than a nutrient transporter, highlighting its broader role in cell envelope stability. This dual function has important implications for understanding how mycobacteria balance impermeability with metabolic needs and suggests new strategies to enhance antibiotic efficacy by targeting membrane-associated proteins like PPE51.

## INTRODUCTION

*Mycobacterium tuberculosis* (Mtb), the pathogen responsible for human tuberculosis, is protected by a highly impermeable and hydrophobic cell envelope. This distinctive feature inherently confers resistance to various toxic compounds and antibiotics, complicating treatment strategies (1, 2). This unique mycobacterial cell envelope, differing from other diderm bacteria such as their gram-negative counterparts, is primarily composed of a number of exclusive lipids, most notably mycolic acids (2–4). Mycolic acids are long fatty acid chains that are either linked to trehalose or the underlying arabinogalactan layer beneath the outer membrane, collectively forming a rigid and impermeable membrane (5–7).

To export proteins across this distinctive cell envelope, mycobacteria have acquired a specialized secretion system called the type VII secretion system (T7S) (8). Mtb possesses five T7S, denoted as ESX-1 to ESX-5, and each of these systems plays a defined role, crucial for the biology and virulence of this pathogen (9, 10). The ESX-5 system, characteristic of slow-growing pathogenic species, has been linked to nutrient transport and is essential for viability (11). It was demonstrated that ESX5 essentiality could be rescued by increasing the membrane permeability in the slow-growing fish pathogen *Mycobacterium marinum* (11). Thus, the ESX-5 system and its secreted substrates might be pivotal in maintaining this remarkably impermeable envelope while simultaneously satisfying the nutritional requirements of these bacteria.

ESX-5 is only present in slow-growing mycobacteria, where this system is responsible for the secretion of most PE and PPE proteins (12). In Mtb, the *pe*/*ppe* genes account for about 10% of the genome coding capacity (9, 13, 14). They received their name from the N-terminal conserved motifs proline-glutamate (PE) and proline-proline-glutamate (PPE) located in the first 10 amino acids (9, 15, 16). Both these classes of proteins have conserved N-terminal domains of 100 and 180 amino acids, respectively. The C-terminal region is more variable in sequence and length and is likely where the specific function resides (15, 16). Like other type VII substrates, PE and PPE proteins are often co-secreted as heterodimers (15, 17). The most reported role of these unique proteins is associated with virulence and immune modulation (16). However, recent research indicates that some of the PEs/PPEs proteins could potentially be involved with nutrient uptake in slow-growing mycobacteria (11, 18–20).

For instance, the heterodimer PE15/PPE20 has been associated with calcium import for maintaining Ca^2+^ homeostasis, and both PPE36 and PPE62 are required for heme scavenging, the major iron reservoir found in the human host (18, 19). Furthermore, in a study by Wang et al., several PEs and PPEs protein pairs with distinct functions were identified. The dimer formed by PE20/PPE31 was reportedly required for magnesium uptake, whereas PE19/PPE25 and PE32/PPE65 were shown to be involved in phosphate influx (20). However, thus far, the most convincing data have probably been obtained with the PE19/PPE51 dimer. Mutants of PE19/PPE51 displayed impaired growth *in vitro* when glucose or glycerol served as the sole carbon source (20). In addition, another study by Korycka-Machała et al. observed that the uptake of disaccharides is linked to PPE51 protein (21). Based on these findings, the PPE51 protein could be the primary transporter of carbohydrate-derived carbon in Mtb. Interestingly, the *ppe51* mutants showed deficient growth during starvation for specific nutrients. Furthermore, these mutant bacteria were able to complement the growth defect by increasing outer membrane permeability, facilitated by compensatory mutations in outer membrane lipid synthesis (20). Thus, there is a strong connection between the impermeability of the membrane and the presence of PPE51. This is very similar to the ESX-5 system, where compensatory mutations enhancing outer membrane permeability or the introduction of the MspA pore also countered the essentiality of ESX-5 (11).

In this study, we further characterized the PPE51 protein by utilizing the tuberculosis model, *M. marinum*. We studied the role of nutrient uptake and cell envelope permeability and established that PPE51 is an ESX-5 substrate. Our results offer a more complete but also more complex understanding of the significance of ESX-5 and PPE51 in the context of nutrient uptake and membrane integrity in pathogenic mycobacteria.

## RESULTS

### PPE51 is involved in glucose and glycerol uptake in *M. marinum*

**Fig 1:**
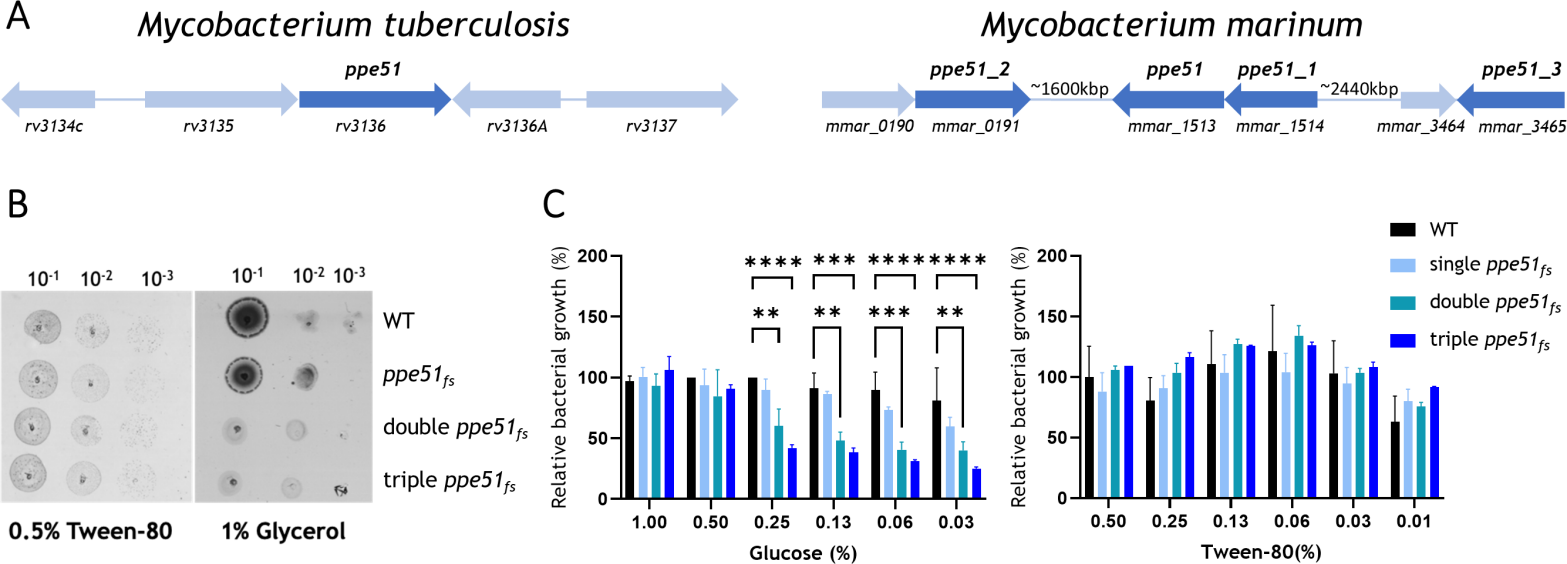
PPE51 is involved in glucose and glycerol uptake in *M*. *marinum*. (**A**) Genomic organization of the *ppe51* (*rv3136*) from *M. tuberculosis* and *ppe51*, *ppe51_1*, *ppe51_2*, and *ppe51_3* from *M. marinum*, all shown in dark blue. (**B**) Growth experiments on solid media, wild-type (WT) and *ppe51_fs_*, *ppe51_fs__ppe51_1_fs_* (double), and *ppe51_fs__ppe51_1_fs__ppe51_2*_fs_ (triple) mutants. Bacteria were dropped on Hartmans-de Bont agar media containing either Tween-80 or glycerol as the sole carbon source. Certain artifacts from the scanner may appear as horizontal and vertical lines in this image. (**C**) Growth experiments in liquid minimal media showing relative bacterial growth (%) at varying concentrations of glucose and Tween-80 as sole carbon sources. Statistical analysis was performed using two-way analysis of variance, with multiple comparisons conducted relative to the WT strain. Statistical significance is indicated by asterisks: *, *P* < 0.05; **, *P* < 0.01; ***, *P* < 0.001; and ****, *P* < 0.0001. Bars represent the mean of two technical replicates; error bars indicate the standard deviation. Data are representative of three independent experiments.

PPE51 plays a significant role in nutrient uptake in Mtb, namely in the uptake of glucose and glycerol, alongside other disaccharides (20–22). Interestingly, the genome of Mtb encodes only one *ppe51* gene (*rv3136*), whereas *M. marinum* has four different paralogs located in various positions on the genome. The genes *mmar_1513*, *mmar_1514*, *mmar_0191*, and *mmar_3465* encode proteins PPE51, PPE51_1, PPE51_2, and PPE51_3, respectively (Fig. 1A). These putative proteins all share a high sequence identity (more than 68% identity), which does not allow the identification of a clear ortholog (Table S1). Therefore, we created frameshift mutants of all four *M. marinum ppe51* genes using a CRISPR-Cas9 editing approach described previously (23). Using these mutants and constructs, we also obtained double and triple *ppe51* mutants (*ppe51*_fs_, *ppe51*_fs__*ppe51_1*_fs_, and *ppe51*_fs_*ppe51_1*_fs__*ppe51_2*_fs_). Interestingly, while single, double, and triple deletions were possible, attempts to delete all four *ppe51* genes were unsuccessful, suggesting that the presence of at least one complete *ppe51* gene is essential for *M. marinum*. To confirm this hypothesis, we transformed the triple *ppe51*_fs_ mutant with a plasmid expressing the *pe19*_mmar_-*ppe51*_mmar_ pair (*mmar_2673*, *mmar_1514*). In this triple mutant background, carrying the *pe19*_mmar_-*ppe51*_mmar_ expression construct, we could successfully create a chromosomal quadruple mutant, supporting the assumption that the presence of at least one *ppe51* copy is essential (Fig. S1). We also attempted to delete the fourth *ppe51* copy in a strain overexpressing *mspA*, following a strategy similar to that previously used to bypass ESX-5 essentiality (11). However, this approach was unsuccessful (data not shown).

Subsequently, we examined the growth of various *ppe51* mutants (single, double, and triple) using different carbon sources (Fig. 1B). The bacteria were grown on defined minimal Hartmans-de Bont (HdB) agar plates, which allowed us to add sole carbon sources selectively, either glycerol or Tween-80. As a positive control, the HdB medium was supplemented with three carbon sources: glucose, glycerol, and Tween-80. Tween-80 is degraded extracellularly by *M. marinum*, and the acyl chains can be utilized as a carbon source. The growth assay experiments showed that both wild-type (WT) and single *ppe51* mutant strains grew equally well on glycerol, indicating that the deletion of one *ppe51* gene alone was insufficient for phenotypical changes on a solid medium. However, when two or more *ppe51* genes were deleted, the ability of *M. marinum* to grow on glycerol as the sole carbon source was impaired. This was not the case when Tween-80 was used as the sole carbon source. These results imply that these mutants do not have a general growth defect but rather a specific deficiency in glycerol uptake.

We next performed additional growth assays using glucose as a sole carbon source (Fig. 1C). The bacterial strains were grown in liquid HdB media supplemented with different concentrations of glucose (0, 0.03%–1%) or Tween-80 (0, 0.02%–0.5%). Deleting two or more *ppe51* genes resulted in impaired growth on glucose as a single carbon source, as shown by the double and triple *ppe51* mutants' growth, which was significantly hampered on glucose. The growth defect of double and triple *ppe51* mutants was not observed when Tween-80 was present in the medium. We also tested other carbon sources and various nitrogen sources; however, the *ppe51* mutants showed no growth difference in comparison to the WT strain under those conditions (Fig. S2). Collectively, these results indicate that PPE51 proteins are involved in glucose and glycerol uptake in *M. marinum*. This means that we could reproduce the previously observed phenotype in Mtb; however, in *M. marinum*, this function appears to be shared among four PPE51 homologs, which seemingly perform similar or identical roles.

### PPE51 is secreted through the ESX-5 secretion system

**Fig 2:**
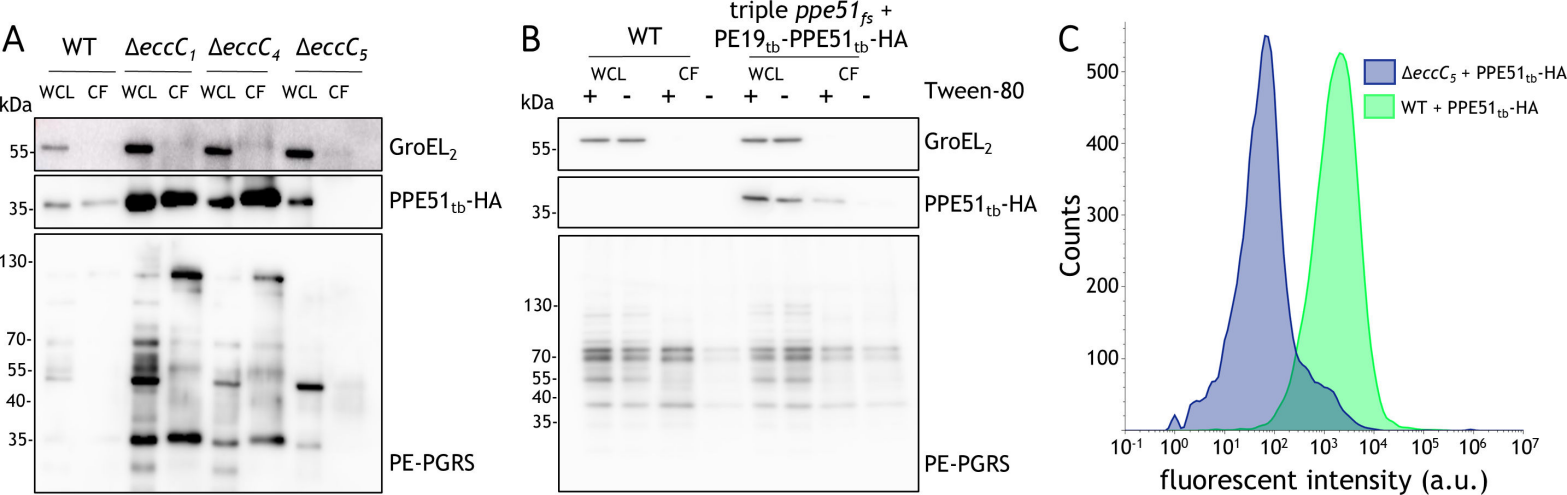
PPE51 is a soluble substrate of the ESX-5 secretion system. (**A**) Secretion analysis of supernatant proteins from WT and ESX-1, ESX-4, and ESX-5 mutant strains lacking the core component EccC, all expressing PPE51tb-HA (*pSMT3-pe19tb-ppe51tb-HA*). GroEL2, a cytoplasmic protein, served as a lysis control, while PE-PGRS proteins were used as a secretion control. WCL refers to whole-cell lysate, and CF denotes culture filtrate. (**B**) Secretion analysis of WT and the triple *ppe51* mutant, both expressing PPE51_tb_-HA. Bacteria were cultured in 7H9-ADS medium with or without Tween-80. Controls are the same as in panel A. (**C**) Flow cytometry analysis of WT and ∆*eccC5* mutant strains to assess PPE51_tb_-HA surface exposure. Detection was performed using an anti-HA primary antibody, followed by a fluorescent Alexa 647-conjugated secondary antibody (goat anti-mouse). The histogram shows the number of cells (*y*-axis) plotted against fluorescence intensity (*x*-axis; arbitrary units, a.u.). The mean fluorescence intensity was 223 for the ∆*eccC5* mutant, compared to 3,094 for the WT, both strains expressing PPE51tb-HA (*pSMT3-pe19tb-ppe51tb-HA*).

PPE proteins are mostly secreted by ESX type VII secretion systems (12). We set out to determine which ESX system is responsible for the secretion of PPE51 (Fig. 2A). We translationally fused the *ppe51*_tb_ gene to an HA tag at the C-terminus and transcriptionally fused this construct with its PE-partner *pe19* (pSMT3-PE19_tb_-PPE51_tb_-HA, referred to as PE19_tb_-PPE51_tb_-HA). Subsequently, this construct was brought into individual mutants lacking functional ESX-1, ESX-4, and ESX-5 systems due to different mutations in the central *eccC* genes. The ESX-2 system is not present in the *M. marinum* M strain, and ESX-3 is essential for the bacterium’s survival; attempts to create a mutant have been unsuccessful (Ummels et al., unpublished results). Secretion analyses were performed by precipitating the secreted proteins in the culture supernatant as described previously (24). As controls, we used antiserum directed against GroEL2 as an intracellular protein reporting on bacterial lysis, and the ESX-5-dependent PE_PGRS proteins were used as a secretion control. Our results show that WT cells and the ESX-1 and ESX-4 mutant strains could secrete PPE51_tb_, as observed by the presence of a band at approximately 35 kDa. This secretion was abolished in the ESX-5 mutant, confirming that PPE51 is indeed an ESX-5 substrate in *M. marinum*. Furthermore, we also observed reduced amounts of cellular PPE51 in the *eccC_5_* mutant, similar to the reduction of PE_PGRS proteins in this mutant. It is known that substrates do not accumulate intracellularly and are degraded in mycobacteria when the type VII secretion pathway is disrupted (12).

### PPE51 is a protein located on the surface of *M. marinum*

Following the identification of ESX-5 as the secretion system for PPE51, we set out to determine the protein’s location. We hypothesized that PPE51 could either remain associated with the bacterial outer membrane or, like other ESX substrates, reside within the capsule layer or be fully secreted into the culture filtrate. To explore this, we performed subcellular fractionation of bacteria and secretion analysis of the culture filtrate. To this end, we used the triple mutant strain overproducing PE19_tb_-PPE51_tb_-HA to avoid any indirect effects produced by the native PPE51_mmar_ proteins. Slight differences in PPE51 localization were observed depending on the growth conditions, specifically the presence or absence of detergent (Fig. 2B). When the bacteria were cultured in the presence of Tween-80, PPE51 was partly detected in the culture filtrate, whereas no release into the culture supernatant was observed when bacteria were grown without detergent. These findings suggest that PPE51 might be loosely attached to the surface of the bacterial membrane and can be extracted during culturing with mild detergents. To investigate this, we employed flow cytometry to assess the surface exposure of PPE51tb-HA in bacteria cultured without Tween-80. Intact WT cells were incubated with a primary anti-HA antibody, followed by a fluorescently labeled secondary antibody (Fig. 2C). As a control, we included the ESX-5 mutant with the same PE19_tb_-PPE51_tb_-HA construct. The gating strategy used is shown in Fig. S3. As expected, the WT cells overexpressing PE19_tb_-PPE51_tb_-HA showed higher fluorescent labeling as compared to the ESX-5 mutant, demonstrating that PPE51 protein is indeed surface-exposed when detergents are omitted in the culture medium.

**Fig 3:**
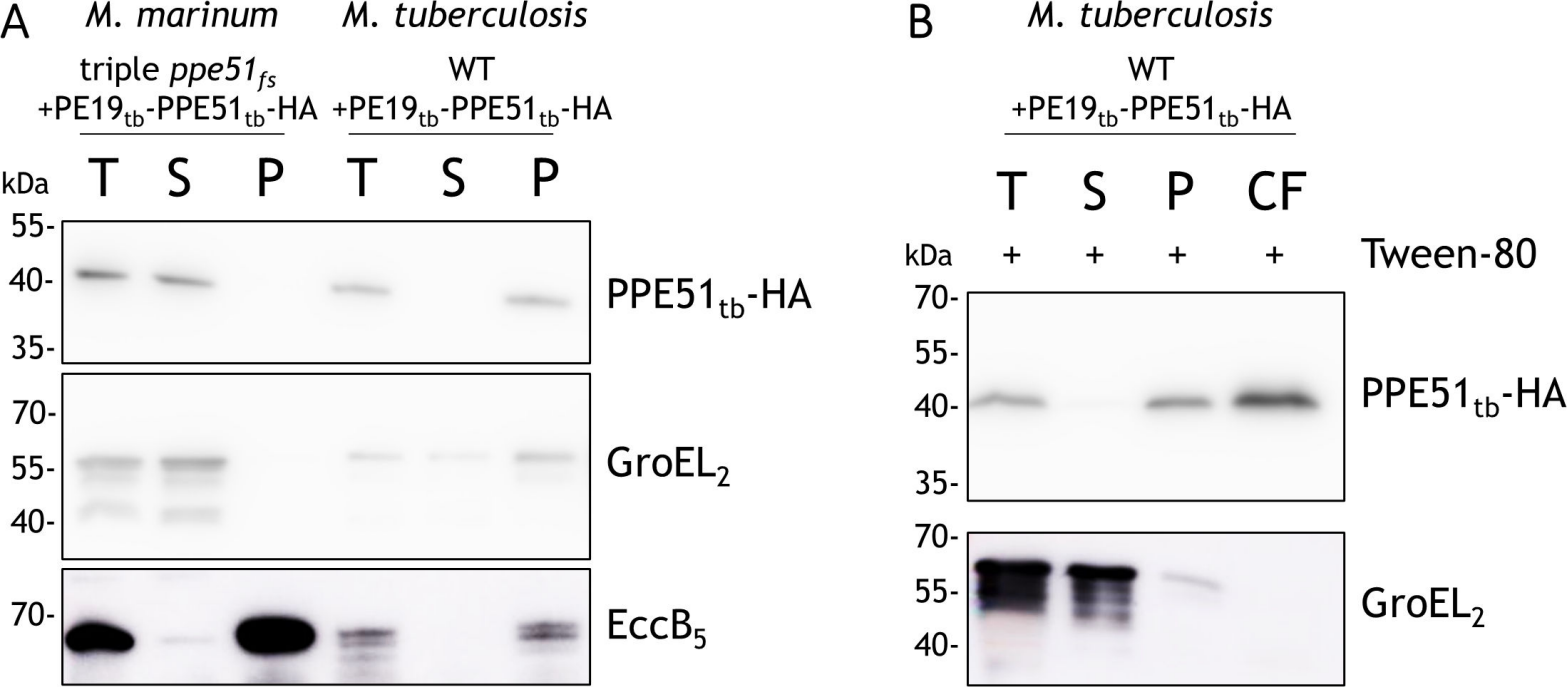
Intracellular localization and secretion analysis of HA-tagged PPE51 in different mycobacterial species. (**A**) Subcellular fractionation of a *M. marinum* triple *ppe51* mutant and *M. tuberculosis*, both overexpressing HA-tagged PPE51_tb_ from the pSMT3 vector. Bacteria were lysed using a French press, and lysates were subjected to high-speed centrifugation to separate soluble (cytoplasmic) and insoluble (membrane-associated) protein fractions. Samples analyzed by immunoblotting included whole-cell lysates (T), soluble fractions (S), and pellet fractions (P). GroEL_2_ served as a lysis control, and EccB_5_, an inner membrane protein conserved in both species, was used as a membrane marker. (**B**) Secretion analysis of *M. tuberculosis* (*pSMT3-pe19tb-ppe51tb-HA*) cultured in the presence of Tween-80. Culture supernatants were collected, and proteins were precipitated for immunoblotting. Bacterial pellets were processed as in panel A to obtain soluble and insoluble fractions. Samples analyzed included whole-cell lysate (T), soluble fraction (S), pellet fraction (P), and culture filtrate (CF). GroEL_2_ served as a lysis control.

To further determine the localization of the fraction of PPE51 that was extracted from the cell surface with mild detergents, we fractionated the triple mutant overexpressing HA-tagged PPE51, together with its PE19 partner. The results indicate that PPE51 is predominantly found in the soluble protein fractionation, with only a small amount detected in the insoluble fraction, corresponding to membrane proteins (Fig. 3A). This result is in contrast to previous experiments performed with Mtb, where PPE51 was mainly identified in the cell wall (20). Therefore, we also performed localization experiments in Mtb. Indeed, the localization of PPE51 in Mtb deviates from the pattern observed in *M. marinum* and was predominantly detected in the insoluble fraction rather than the soluble fraction (Fig. 3A). However, secretion analysis of this strain, conducted in the presence of Tween-80, revealed a phenotype similar to that observed in *M. marinum*, i.e., a substantial portion of PPE51 is detected in the culture filtrate. These findings suggest that, while the subcellular localization of PPE51 appears to vary between mycobacterial species, its extractability by mild detergents indicates that it is similarly surface-associated in both *M. marinum* and *Mtb* (Fig. 3B).

### Complex formation of PPE51 on the bacterial surface

The predicted AlphaFold structure of PPE51 shows a well-defined N-terminal bundle of helices typical of a PPE protein. This domain is required for secretion and probably does not play a role in the function of the protein, as it is often removed by surface proteases such as PecA (25). The functional C-terminal domain of PPE51 is undefined by AlphaFold (PDB: P9WHY3) (Fig. S4). Because the C-terminal domain is also relatively small, we hypothesized that, in order to function as a transporter, PPE51 should form higher molecular-weight complexes in the mycobacterial cell envelope. We therefore investigated the potential of PPE51 to form complexes. To this end, we used the triple mutant expressing HA-tagged PPE51_tb_, together with its PE19_tb_ partner. The proteins were extracted with different mild detergents to preserve a possible protein complex, including n-dodecyl-β-D-maltoside (DDM), β-octyl, Genapol X-080, Tween-80, Zwittergent, tyloxapol, Triton X-100, and CHAPS, and compared the protein extraction efficiency (Fig. S5A). Sodium dodecyl sulfate (SDS) and phosphate-buffered saline (PBS) served as positive and negative controls, respectively. Our findings revealed that several detergents could extract PPE51 in sufficient quantities, as indicated by the presence of a band corresponding to PPE51_tb_-HA. Zwittergent and tyloxapol demonstrated the least effective PPE51 extraction compared to the other detergents (DDM, β-octyl, Genapol X-080, Tween-80, Triton X-100, and CHAPS). Surprisingly, the negative control (PBS) also contained small amounts of PPE51, which may be the result of protein detaching from the membrane. Based on this, we selected Genapol X-080 and Triton X-100, two non-ionic detergents, as our preferred detergents.

To assess the oligomeric native state, PPE51_tb_-HA was extracted, separated according to size using blue-native (BN) gel electrophoresis, and detected by immunoblotting (Fig. S5B). To avoid interference with the native protein electrophoresis, detergents used during the extraction were at low concentrations. The concentrations used were 0.06%, 0.1%, and 0.3% for both detergents. This corresponds to 5, 8, and 23 times the critical micelle concentration (CMC) value of Triton X-100 and 8, 12, and 38 times the CMC value of Genapol X-080. Genapol X-080 and Triton X-100 successfully extracted PPE51 even at the lowest tested concentration, suggesting that PPE51 is not firmly attached to the mycobacterial outer membrane. In addition, we observed an unresolved signal at a higher molecular weight, around 720 kDa, and more discrete bands around 146 kDa. These observations provide evidence that PPE51 indeed forms complexes on the surface of *M. marinum* bacteria. However, additional experiments must be performed to investigate whether these complexes are homomultimers or include additional proteins.

### Deletion of PPE51 results in increased membrane permeability of *M. marinum*

**Fig 4:**
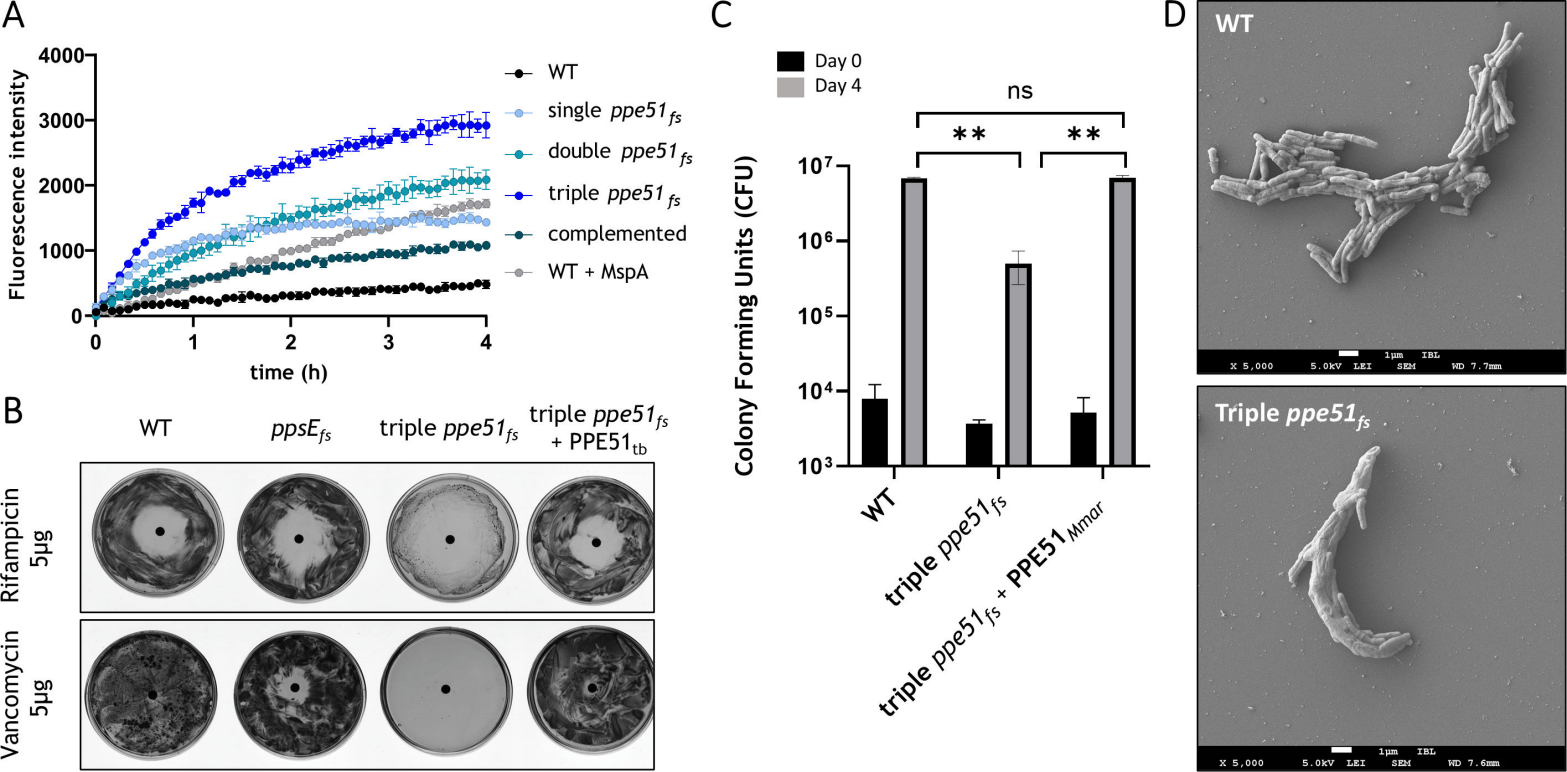
Role of PPE51 in membrane permeability and integrity. (**A**) EtBr uptake in the *ppe51_fs_* (single *ppe51_fs_*), *ppe51_fs__ppe51_1_fs_* (double *ppe51_fs_*), and *ppe51_fs__ppe51_1_fs__ppe51_2_fs_* (triple *ppe51_fs_*) strains, as well as in the complemented strain *ppe51_fs__ppe51_1_fs__ppe51_2_fs_* expressing pSMT3-*pe19*_mmar_-*ppe51*_mmar_. Control strains include the WT and WT overexpressing MspA as a positive control. Bacteria were incubated with 5 µg/mL EtBr, and fluorescence was measured over time to assess membrane permeability. Data shown are representative of three independent experiments, and the error bars represent the standard deviation of four technical replicates. (**B**) Antibiotic susceptibility assay of the triple *ppe51_fs_* mutant and the complementation strain, with WT and the phthiocerol dimycocerosate (PDIM)-deficient *ppsE_fs_* mutant as controls. Bacteria were grown on 7H10 agar plates, and susceptibility was determined using a disk diffusion assay with vancomycin (5 µg) and rifampicin (5 µg). Inhibition zone diameters were measured to assess antibiotic susceptibility (rifampicin: WT, 13.96 mm; triple *ppe51_fs_*, 29.27 mm; *ppe51_fs_ +* PPE51_Mtb_, 21.48 mm; *ppsE_fs_*, 26.65 mm and vancomycin: WT, no inhibition zone; triple *ppe51_fs_*, 15.53 mm; *ppe51_fs_ +* PPE51_Mtb_, 5.92 mm; *ppsE_fs_*, no inhibition zone). (**C**) Macrophage infection using the murine macrophage cell line J774A.1. Macrophages were infected with WT, the triple *ppe51_fs_* mutant, and the complementation strain at a multiplicity of infection of 1. After 3 hours (Day 0) and 4 days post-infection (Day 4), macrophages were lysed and plated for CFU counting. Statistical analysis was performed using two-way analysis of variance, with multiple comparisons conducted relative to the WT strain. The difference between WT and the triple *ppe51_fs_* mutant was statistically significant (adjusted *P* = 0.005), as was the difference between the mutant and the complementation strain (*P* = 0.0047). (**D**) Scanning electron microscopy (SEM) images of *M. marinum* WT and the triple *ppe51_fs_* mutant.

Given that PPE51 is a surface-exposed protein involved in nutrient acquisition and associated with the membrane, we investigated whether the absence of PPE51 could potentially affect the overall permeability of the mycobacterial cell envelope (16). To test this, we performed an ethidium bromide (EtBr) uptake assay, which serves as an indicator of membrane permeability (Fig. 4A) (26). Ethidium bromide is a dye whose fluorescence drastically increases upon DNA binding, allowing uptake measurements over time. PPE51 frameshift mutants were incubated with ethidium bromide and monitored for uptake over several hours. The WT strain and a WT strain overexpressing the MspA porin from *Mycobacterium smegmatis* served as negative and positive controls, respectively. Overexpression of the MspA porin has been shown by Ates et al. (11) to increase the membrane permeability of *M. marinum*. Surprisingly, our results revealed that all three *ppe51* mutants (single, double, and triple) showed a significantly increased ethidium bromide uptake. Specifically, we observed that the deletion of a single *ppe51* gene already led to an increase in ethidium bromide accumulation, but after subsequent deletion of the second and third copy, membrane integrity was severely compromised, to levels higher than those of the positive control. These results indicate that, although the uptake of glycerol and glucose is impaired, the uptake of other small molecules is highly increased.

### Increased permeability results in higher susceptibility toward high-molecular-weight drugs

The mycobacterial membrane is a known bottleneck for antimicrobial compounds (1). This membrane acts as the first line of defense by preventing antibiotics from entering the cell and thus rendering antibiotics that target intracellular proteins ineffective against Mtb (27). However, our observation of increased permeability in our *ppe51* mutants prompted us to investigate their susceptibility to high-molecular-weight antibiotics (Fig. 4B). We selected the cell-wall synthesis inhibitor vancomycin (1,486 g/mol), which is known to be ineffective against mycobacteria because it is unable to penetrate the mycobacterial outer membrane. We also included rifampicin, which has a high molecular weight (823 g/mol), but whose target is in the bacterial cytosol (28–30). To assess the antimicrobial susceptibility of the *ppe51* triple mutant compared to wild-type *M. marinum*, we performed disc diffusion assays. Mid-logarithmic phase cultures were adjusted by optical density and uniformly spread onto 7H10 agar plates. Filter paper discs containing rifampicin or vancomycin were then placed on the agar surface. Following incubation, zones of inhibition were measured to evaluate the differential susceptibility between the strains. We included the phthiocerol dimycocerosate (PDIM)-deficient *ppsE* mutant as a positive control and the PPE51 complementation strain, expressing *pe19*_tb_ and *ppe51_tb_* in the triple frameshift *ppe51* mutant (pSMT3-PE19_tb_-PPE51_tb_, referred to as PE19_tb_-PPE51_tb_). Wild-type *M. marinum* cells showed no susceptibility to vancomycin, whereas only a small inhibition zone (1.4 cm) was present around the discs with rifampicin. The *ppsE* mutant exhibited a susceptibility to rifampicin comparable to the WT strain, with only a marginally increased sensitivity to vancomycin (Fig. 4B). In contrast, the *ppe51* triple mutant demonstrated significantly increased susceptibility to these high-molecular-weight antibiotics. This was evident from the inhibition zones, measuring 2.9 cm for rifampicin and a strikingly large zone for vancomycin, which extended beyond the edges of the agar plate (Fig. 4B). Notably, the susceptibility phenotype in the *ppe51* triple mutant was fully restored to WT levels for both antibiotics in the complemented strain.

### The ppe51 frameshift mutant exhibited impaired intracellular growth within macrophages

The low permeability of the mycobacterial outer membrane plays a crucial role in protecting the bacteria against host effector molecules, including antibacterial proteins and toxic compounds, during infection. We hypothesized that the increased membrane permeability observed in the absence of PPE51 could compromise the protective integrity of the cell envelope, thereby reducing mycobacterial virulence. To test this, we examined the behavior of the *ppe51* triple mutant strain during macrophage infection (Fig. 4C). We infected J774A.1 murine macrophage cells with *M. marinum* WT, the triple frameshift *ppe51* mutant, and the complemented strain. We next monitored the infections for a period of 4 days and evaluated virulence by determining the bacterial load. Our findings show that the *ppe51* triple frameshift mutant exhibits significantly reduced intracellular growth within macrophages at 4 days post-infection, compared to the wild-type strain. This attenuation in virulence was reversed upon complementation with *pe19_mmar_-ppe51_mmar_,* restoring intracellular growth rates to wild-type levels. To further investigate the reason for the attenuated phenotype observed in the triple *ppe51_fs_* mutant, we tested its response to different host-related stress factors, namely varying pH levels and susceptibility to antimicrobial enzymes such as lysozyme (Fig. S6). The results showed no clear phenotype for the mutant, which in both cases resembled the WT cells.

### Membrane morphology changes in the triple *ppe51* frameshift mutant

To better understand the role of *ppe51* in mycobacterial membrane integrity, we analyzed the membrane morphology in detail, as structural changes in the cell envelope might contribute to the altered permeability and susceptibility phenotypes observed in the *ppe51* mutants. Using scanning electron microscopy, we compared the cell membrane structure of the *ppe51* triple mutant to that of wild-type *M. marinum* cells (Fig. 4D; Fig. S7).

Our results revealed notable differences in cell morphology upon deletion of the three *ppe51* gene copies. The characteristic cording of cells formed by *M. marinum* appeared altered in the mutant strain, with individual cells displaying a more fused and less distinct morphology compared to the well-defined boundaries of wild-type cells within the cording cell aggregates. This altered morphology probably reflects changes in the organization of the cell envelope, potentially contributing to the observed increase in permeability to ethidium bromide and enhanced sensitivity to high-molecular-weight antibiotics.

While the exact relationship between these morphological changes and functional alterations remains unclear, these findings highlight the critical role of *ppe51* in maintaining the structural and functional integrity of the mycobacterial cell envelope and in the characteristic shape of cording *M. marinum*. Further studies are needed to dissect the molecular mechanisms underlying these phenotypes.

## DISCUSSION

In this study, we provide new insights into the potential roles of PPE51 in maintaining membrane integrity and facilitating nutrient uptake in pathogenic mycobacteria. Additionally, we identified PPE51 as a substrate of the ESX-5 secretion system, which supports its proposed involvement in nutrient transport across the outer membrane (11). While these findings advance our understanding, the precise molecular mechanisms underlying PPE51’s contributions remain to be elucidated.

Different from Mtb, *M. marinum* contains four paralogs of *ppe51* located at three genomic regions. Therefore, we constructed mutants lacking the individual genes and also double and triple mutants. To our surprise, several attempts failed to create the quadruple mutant. This desired mutation in the fourth *ppe51* gene was only achieved when an intact copy of *ppe51* was included on a plasmid. From this, we can conclude that the presence of at least a single copy of *ppe51* is essential in *M. marinum*, which is different from Mtb. Interestingly, we were unable to generate the quadruple mutant in a strain expressing *mspA*. This was unexpected, as PPE51 is an ESX-5 substrate, and *mspA* expression has previously been shown to bypass ESX-5 essentiality (11). To address this in more detail, a conditional expression system as described by Kim et al. (31) may provide a suitable approach for generating knockdown mutants of essential genes. Notably, when looking at previous whole-cell proteomics data, we observed that not all copies of *M. marinum*’s PPE51 proteins are produced, as PPE51_1 is the only one to be detected (11). This could indicate that the paralogs serve as redundant copies. Given that PPE51 is an ESX-5 substrate involved in nutrient acquisition and membrane integrity, it is tempting to speculate that PPE51 might be a key factor contributing to the essentiality of the ESX-5 system.

The variation in the number of *ppe51* copies between *M. marinum* and Mtb may be attributed to the distinct environmental niches these bacteria inhabit. The facultative pathogen *M. marinum* grows in diverse environmental conditions and necessitates greater flexibility in utilizing various carbon sources. In contrast, Mtb, as an obligate pathogen adapted to the human host, can afford to specialize its metabolism to meet the specific requirements of its host environment (32). Moreover, Mtb is recognized for its metabolic plasticity, showcasing the ability to adapt to various nutritional sources (33). Mtb displays a preference for cholesterol and fatty acids as primary carbon sources, aligning with its specialized adaptation to the unique metabolic landscape within the mammalian host (34–36). Little is known about *M. marinum* metabolic plasticity. A possible explanation for the presence of these four variants is that PPE51 may play a broader role, potentially involving a wider range of substrates in processes related to nutrient acquisition in facultative pathogenic mycobacteria.

Based on our results, we found that PPE51 is loosely associated with the bacterial surface of *M. marinum*, as it could be readily washed off when the bacteria were grown in a medium containing mild detergents. This strongly suggests that PPE51 is not an integral outer membrane transport channel, like porins, but rather a surface-associated protein. However, we were still able to link its role to nutrient uptake as previously described (20–22). Thus, we hypothesize that, in *M. marinum*, PPE51 facilitates nutrient transport across the outer membrane through a mechanism that might not involve stable pore formation. One possible explanation is that PPE51 may have a regulatory role at the membrane, potentially influencing the localization or stability of other membrane-associated proteins. However, we currently lack data to support this, and further research will be needed to investigate this possibility more directly. What is clear is that PPE51 remains loosely attached to the outer surface of the cell envelope after nutrient transport, rendering it detergent-extractable. However, as neither glucose nor glycerol represents the natural or dominant carbon source of mycobacteria (26, 37), whether the uptake of those carbon sources reflects the physiological function of PPE51 or an experimental artifact remains to be further investigated.

Interestingly, although the subcellular localization of PPE51 in Mtb differs (being primarily found in the insoluble fraction) (20), the same hypothesis may still be relevant. This is because, even in Mtb, when grown with Tween-80, a considerable amount of PPE51 is detected in the culture filtrate (Fig. 3B), suggesting a non-porin-like characteristic. Supporting this, PPE51 has been detected in the Triton X-114 extractable fraction by Målen et al. (38), which is enriched for membrane and membrane-associated proteins, consistent with our localization data. In contrast, de Souza et al. (39, 40) did not identify PPE51 among the proteins present in the culture filtrate of Mtb H37Rv, detecting it only in the membrane fraction. This discrepancy is likely due to differences in the growth conditions, as their culture filtrate was derived from bacteria grown as a surface pellicle without detergent. This still aligns with our hypothesis that PPE51 is membrane-associated and can only be detected in the culture filtrate when detergents are present.

By deleting PPE51 paralogs, we initially expected to reduce the permeability of the *M. marinum* cell wall to nutrients and other compounds. However, when we tested the ethidium bromide uptake in the mutant cells, we observed a rapid and substantial accumulation. Surprisingly, this suggests that deleting PPE51 makes the membrane more permeable to other compounds. Notably, in our experiments, complementation restored the wild-type phenotype, indicating that the deletion did not disrupt PDIM integrity. This contrasts with previous studies in Mtb, where *ppe51* deletion led to PDIM loss, complicating phenotypic analyses (20). The observed increase in membrane permeability in the *ppe51* mutant suggests a dual role for PPE51: in addition to facilitating nutrient transport across the myco-membrane, it may also remain in the outer leaflet of the outer membrane to contribute to maintaining membrane integrity.

Scanning electron microscopy supports this hypothesis since it revealed clear alterations in the bacterial membrane and changes in morphology in cording cells of the triple *ppe51* mutant, which could be the result of or reason for the loss of membrane integrity. In line with this loss of membrane integrity, the triple *ppe51* mutant was found to be attenuated in macrophages as well as more susceptible than WT to high-molecular-weight antibiotics such as vancomycin and rifampicin. Mycobacteria are intrinsically resistant to vancomycin, which cannot penetrate the myco-membrane, but are sensitive to rifampicin, which is part of the short-course combination therapy for tuberculosis treatment. Antibiotic susceptibility phenotypes are commonly linked to modifications in the membrane structure, particularly when the observed effects are seen with antibiotics targeting diverse cellular components (27).

Nevertheless, a puzzling observation is that cells with increased permeability, which are now susceptible to large antibiotics, cannot utilize glycerol, a small molecule that, in principle, should penetrate the more permeable cell wall. This discrepancy highlights our limited understanding of the impermeable outer mycolic membrane. One possible explanation is that the increased ethidium bromide uptake may reflect not only a leakier membrane but also altered surface charges, differentially affecting the uptake of charged versus uncharged compounds like glucose and glycerol. Alternatively, the apparent increase in permeability might be overestimated; while even a small number of antibiotic molecules can enter and kill the bacteria, the uptake of nutrients like glucose or glycerol as sole carbon sources requires a significantly higher influx. This difference may explain why the mutant can take up large antibiotics but fails to utilize small nutrients for growth.

Our findings suggest that the mechanisms governing permeability and nutrient transport in mycobacteria are more complex than previously thought. Further research is essential to unravel how transport is achieved across the mycobacterial membrane, which may hold the key to understanding both nutrient uptake and antibiotic resistance in these bacteria. In conclusion, PPE51 proteins are surface-exposed membrane proteins that assist in glucose and glycerol transport but also affect the integrity of the mycobacterial outer membrane.

## MATERIALS AND METHODS

### Bacterial strains and growth conditions

*M. marinum* M^USA^ was used as the parental strain for all mutants (Table S2). *M. marinum* strains were routinely grown at 30°C in liquid Middlebrook (Difco-BD Biosciences) 7H9 or solid Middlebrook 7H10 medium supplemented with 10% (vol/vol) of ADS (5% [wt/vol] albumin, 2% [wt/vol] dextrose, and 0.16% [wt/vol] NaCl), 0.2% glycerol, and 0.02% (vol/vol) tyloxapol. *Escherichia coli* Dh5α, used for plasmid construction, was grown in Luria-Bertani liquid and solid medium at 37°C. If necessary, antibiotics kanamycin (Kan, 25 µg/mL; Sigma) and hygromycin (Hyg, 50 µg/mL; Roche) were added to the media.

### Molecular cloning for frameshift mutants

All primers and oligos to construct small-guide RNAs (sgRNAs) within this study can be found in Table S3. The CRISPR-Cas9 cloning technique created frameshift mutations in selected genes. The target site prediction and the generation of mutants were performed as described previously (23). Briefly, the CRISPR plasmid pCRISPRx-Sth1Cas9-L5, containing sgRNA targeting *mmar_1513*, *mmar_1514*, *mmar_0191*, and *mmar_3465* genes, was transformed into *M. marinum* on plates containing 200 ng/mL of anhydrotetracycline (IBA Life-sciences) to induce the expression of the *cas9*. The resulting colonies were analyzed by amplifying (PCR) the targeted gene and sequencing the PCR product to verify mutants. All mutant strains are listed in Table S2. The plasmid description can be found in Table S4.

### Molecular cloning of PPE51 expression plasmids

All plasmids obtained in this study are listed in Table S4. The CRISPR deletion vectors were constructed by insertion of hybridized oligo pairs (Table S3), encoding the gene-specific guide RNAs, into the backbone pCRISPRx-Sth1Cas9-L5 as described previously (23). To obtain the expression vectors pSMT3-PE19_tb_-PPE51_tb_ and pSMT3-PE19_mmar_-PPE51_mmar_, the genes *pe19_tb_* and *pe19_1_mmar_* were first amplified (PCR) from chromosomal DNA of Mtb H37Rv and *M. marinum* M^USA^ with the primer pair V01/V02 and V03/04 (Table S5), respectively. The amplified PCR products and the plasmid pSMT3::*mspA* were digested using the restriction enzymes NheI and BamHI. The resulting backbone of pSMT3::*mspA* was ligated with the digested PCR products to yield the plasmids pSMT3-*pe19_tb_* and pSMT3-*pe19_mmar_*. The *ppe51_tb_* gene (*rv3136*) and *ppe51_1_mmar_* gene (*mmar_1514*) were amplified by PCR using primer pairs V05/V06 and V07/V08, respectively, digested with BamHI and XbaI, and ligated into the backbone pSMT3-*pe19_tb_* and pSMT3-*pe19_mmar_*, which were digested with the same enzymes, to yield the plasmids pSMT3-*pe19_tb_- ppe51_tb_* and pSMT3-*pe19_mmar_- ppe51_mmar_*. To obtain a PPE51_tb_-HA-tagged construct, we performed two subsequent extension PCRs. The product of the first amplification with primer pair V01/V02 using chromosomal DNA of Mtb served as the template for the second reaction with primer pairs V09/V10 to yield an amplicon encoding for *ppe51_tb_* translationally fused to an HA-tag. The backbone pSMT3-*pe19_tb_-ppe51_tb_* was digested with SpeI and XbaI and received the amplified *ppe51_tb_HA* gene using recombinational cloning (In-Fusion, Takara Bio) to yield the plasmid pSMT3-*pe19_tb_-ppe51_tb_HA*.

### Growth assay on solid media

The bacteria were grown in 7H9-ADS-glycerol-tyloxapol liquid medium supplemented with the appropriate antibiotics until they reached the mid-logarithmic phase (OD_600_ = 0.8–1.2). Then, the bacteria were harvested and washed with PBS supplemented with tyloxapol (0.02%) and diluted to an OD_600_ of 0.1. The bacteria were 10-fold serially diluted and dropped on solid HdB medium (41) agar plates (HdB medium with 1% Noble agar) supplemented with a single carbon source.

### Growth assay in liquid media

The resazurin microtiter plate assay (REMA) was used to determine the growth of different mycobacterial strains under different conditions (42). Selected carbon sources were diluted in bacterial growth medium as twofold serial dilutions in 96-well plates. Bacteria were routinely grown until the mid-logarithmic phase, harvested by centrifugation, washed in PBS supplemented with 0.02% tyloxapol, and resuspended in the growth medium and added to the 96-well plates containing dilutions of a carbon source to achieve the final OD_600_ of 0.001 per well. The plates were statically incubated for 4 days at 30°C. After incubation, resazurin solution containing resazurin sodium salt (0.025% wt/vol in milliQ) and 20% Tween-80 (ratio 3:1) was added to each well, and the plates were further incubated. When a color conversion of the dye was observed, the fluorescence was measured using a BioTek plate reader (Synergy H1) in bottom-reading mode, with excitation at 560 nm/emission at 590 nm.

### EtBr uptake experiments

Cells were grown in 7H9-ADS-glycerol-tyloxapol medium supplemented with appropriate antibiotics until they reached the mid-logarithmic phase at OD_600_ = 0.8–1.2. On the day of the experiment, bacteria were washed twice with PBS supplemented with 0.02% tyloxapol. Bacteria were resuspended in PBS and distributed with a final OD_600_ of 0.8 in a transparent, round-bottom, 96-well microtiter plate. EtBr was added to the bacterial suspension to a final concentration of 5 µg/mL. Fluorescence was measured every 3 minutes at 30°C for 3–6 hours (excitation: 300 nm/emission: 605 nm) using a BioTek plate reader (Synergy H1) in bottom-reading mode.

### Protein secretion analysis

Proteins from bacterial culture supernatant were precipitated as performed by Daleke et al*.* (24). *M. marinum* strains were grown until the mid-logarithmic phase in 7H9-ADS-glycerol-tyloxapol liquid medium supplemented with appropriate antibiotics. Bacteria were washed and diluted to an OD_600_ of 0.3 in media without ADS, but with glucose. After 16 hours of growth, cells were harvested (3,000 × *g*, 5 minutes), and the culture supernatant fractions were collected. Supernatants were precipitated by the addition of 100% wt/vol trichloroacetic acid. The bacterial pellet was lysed by bead beating (100 mm silica beads; Biospec) to obtain the whole-cell lysate. Proteins were denatured with SDS, separated by SDS-PAGE, and detected by Western blotting.

### Subcellular fractionation

All subsequent steps were performed at 4°C. Bacteria were lysed by passaging them twice through a high-pressure homogenizer (OneShot) using a pressure of 0.83 kbar. Unbroken cells were removed by centrifugation (5,000 × *g,* 10 minutes), and supernatants were transferred to ultracentrifugation tubes. Ultracentrifugation (100,000 × *g*, 1 hour) separated the soluble and insoluble protein fractions.

### Detergent-based protein extraction

Bacteria were grown in 7H9-ADS-glycerol-tyloxapol medium to mid-logarithmic phase (OD_600_ = 0.8–1.2). The bacteria were harvested by centrifugation (5,000 × *g*, 10 minutes) and resuspended in PBS to a final concentration of 20 OD units per mL. The samples were aliquoted (0.25 mL), centrifuged (17,000 × *g*, 5 minutes), and resuspended in 100 µL of PBS supplemented with indicated detergents. After 30 minutes of incubation with head-over-head rotation at room temperature (RT), the samples were pelleted (17,000 × *g*, 5 minutes). The supernatants, containing detergent-extracted surface proteins, were collected and subjected to protein electrophoresis and immunodetection by Western blot analysis.

### BN-PAGE

Detergent-extracted protein complexes were subjected to BN-PAGE analysis. The native protein samples were separated by size using a 4%–16% NativePage Bis-Tris Protein Gel (Invitrogen) after the addition of NativePage 5% G-250 Sample Additive (Invitrogen). The gels were stained with R-250 (CBB; Bio-Rad) and electroblotted to a polyvinylidene difluoride (PVDF) membrane (GE Healthcare Life Sciences). The subsequent immunodetection steps were identical to the standard Western blot method.

### Western blot analysis

Proteins were separated by SDS-PAGE (12.5% polyacrylamide) and transferred to a nitrocellulose membrane (GE Healthcare Life Sciences). Next, the membranes were blocked for 1 hour at RT with 5% skim milk in phosphate-buffered saline with Tween-20 (PBST). Proteins were labeled by using primary mouse monoclonal antibodies anti-HA.11 (1:5,000), anti-PE_PGRS (1:10,000, 7C4.1F7), and anti-GroEL2 (1:10,000, CS44; John Belisle, NIH, Bethesda, MD, USA), for 1 hour at RT. The secondary antibody, goat anti-mouse IgG (1:2,500; Rockland) conjugated with HRP, was incubated for 1 hour, and proteins were visualized by the addition of enhanced chemiluminescence (ECL) substrate (GE Healthcare Life Sciences).

### Flow cytometry

Bacteria were grown to mid-logarithmic phase (OD_600_ = 0.8–1.2) in 7H9-ADS-glycerol without tyloxapol. They were then diluted to an OD_600_ of 0.6 in 7H9-ADS-glycerol medium without detergents and were let grow overnight. The next day, the bacteria were pelleted, washed with PBS with 1% bovine serum albumin (BSA; Sigma), and incubated for 1 hour at 1:1,000 dilution, with an antibody recognizing the HA tag (2-2.2.14; Thermo Scientific). After washing with PBS with 1% BSA, the bacteria were incubated with the secondary antibody goat anti-mouse IgG (A-21235; 420 ThermoFisher) conjugated to Alexa 647 antibodies and SYTO-9 DNA-staining (S34854; ThermoFisher) for 30 minutes. After washing with PBS with 1% BSA, the bacteria were analyzed by flow cytometry (Attune NxT; ThermoFisher). As a control, bacteria were incubated only with secondary antibodies and only with the SYTO-9 stain.

### Antibiotic susceptibility testing

Bacteria were grown in 7H9-ADS-glycerol-tyloxapol liquid medium until the mid-logarithmic phase (OD_600_ = 0.8–1.2). The bacteria were washed with PBS and plated on 7H10-ADS plates at a final OD of 0.1. Antimicrobial discs of 5 µg rifampicin (Oxoid; Thermo Scientific) and 5 µg vancomycin (Oxoid; Thermo Scientific) were placed in the center of the plates. The plates were incubated at 30°C for 7 days.

### Infection with J774A.1 macrophage

Bacteria were grown until mid-logarithmic phase. Bacteria were then harvested by centrifugation, washed, and passed through a 5 µm syringe filter to remove aggregated cells. The infection stocks of each strain were prepared in Dulbecco’s modified Eagle medium (DMEM) GlutaMAX (Gibco) medium with 10% fetal bovine serum (FBS), supplemented with 20% glycerol, and stored as aliquots at −80°C until use. Murine macrophage cell line J774A.1 (ATCC TIB67) was routinely grown in DMEM GlutaMAX medium with 10% FBS at 37°C in 5% CO_2_. The macrophages were seeded as 2.5 × 10^4^ cells per well in a flat-bottom, transparent 96-well plate to perform the experiment. After incubation, the cells were washed and infected with bacteria at a multiplicity of infection of 1. The infection proceeded for 3 hours at 32°C in 5% CO_2_. The extracellular bacteria were removed by washing and addition of a fresh culture medium containing gentamicin (50 µg/mL) for 1 hour at 32°C. The cells were washed twice and incubated with fresh culture medium at 32°C in 5% CO_2_. At different time points (3 hours or 96 hours after incubation), the infected macrophage monolayers were washed once with PBS and lysed with 200 µL of 0.2% Triton X-100 in PBS (Sigma) for 15 minutes to release the intracellular mycobacteria. The bacteria were quantified by plating 10-fold serial dilutions of lysates on 7H10 plates or HdB plates (1% Noble agar) supplemented with 1% Tween-80 as the sole carbon source. The plates were imaged after the growth of single colonies was visible. Statistical significance was determined with the two-way analysis of variance test between the CFU count of the tested strains on Day 4.

### Scanning electron microscopy

Wild-type and triple *ppe51* frameshift mutant bacteria were grown until mid-logarithmic phase (OD_600_ = 0.8–1.2) in 7H9-ADS-tyloxapol medium. Bacterial cells were fixed for 15 minutes with 1% (vol/vol) glutaraldehyde in PBS at room temperature on poly-L-lysine covered glass slides (15 mm diameter). Samples were washed twice with PBS to remove excess fixative and were subsequently serially dehydrated by consecutive incubations in 70% acetone (vol/vol), 80% acetone (vol/vol), 90% acetone (vol/vol), 96% acetone (vol/vol), and 100% acetone (vol/vol). This was followed by critical point drying of the coverslips in a Bal-Tec CPD 030. After critical point drying, samples were mounted on 12 mm specimen stubs (12 mm; Agar Scientific) and coated with platinum/palladium (80/20) to 20 nm using a Quorum Q150T S sputter coater. Samples were examined at 5 kV at ~8 mm working distance with a JEOL 7600 scanning electron microscope.

## SUPPLEMENTAL MATERIAL

Supplemental figures and tables mbio.01044-25-s0001.docx Figures S1 to S7; Tables S1 to S5.

ASM does not own the copyrights to Supplemental Material that may be linked to, or accessed through, an article. The authors have granted ASM a non-exclusive, world-wide license to publish the Supplemental Material files. Please contact the corresponding author directly for reuse.
