# Supplementary material for: PPE51 modulates membrane integrity in Mycobacterium marinum
Source: mBio. 2025 Sep 22;16(11):e01044-25. doi: 10.1128/mbio.01044-25 (PMC12607722; doi:10.1128/mbio.01044-25)
Supplement: Supplemental figures and tables — Figures S1 to S7; Tables S1 to S5. [file mbio.01044-25-s0001.docx]

**Supplemental Material**

**PPE51 Modulates Membrane Integrity in *Mycobacterium marinum***

Vicky Charitou ^a^, Beatriz Izquierdo Lafuente ^b^, Eva Habjan ^a^, Coen Kuijl ^a^_,_ Joost J. Willemse ^c^, Wilbert Bitter ^a, b^, Alexander Speer ^a,*^

^a^ Department of Medical Microbiology and Infection Control, Amsterdam UMC, Location VU Medical Center, van der Boechorststraat 6, 1081 BT Amsterdam, The Netherlands

^b^ Section Molecular Microbiology, Amsterdam Institute of Molecular and Life Sciences (AIMMS), Vrije Universiteit Amsterdam, De Boelelaan 1108, 1081 HZ Amsterdam, The Netherlands

^c^ Institute of Biology, Leiden University, Sylvius Laboratory, Sylviusweg 72, 2333 BE Leiden, The Netherlands

* **For correspondence:**  [a.speer@amsterdamumc.nl](mailto:a.speer@amsterdamumc.nl)

**Supplemental Figures:**


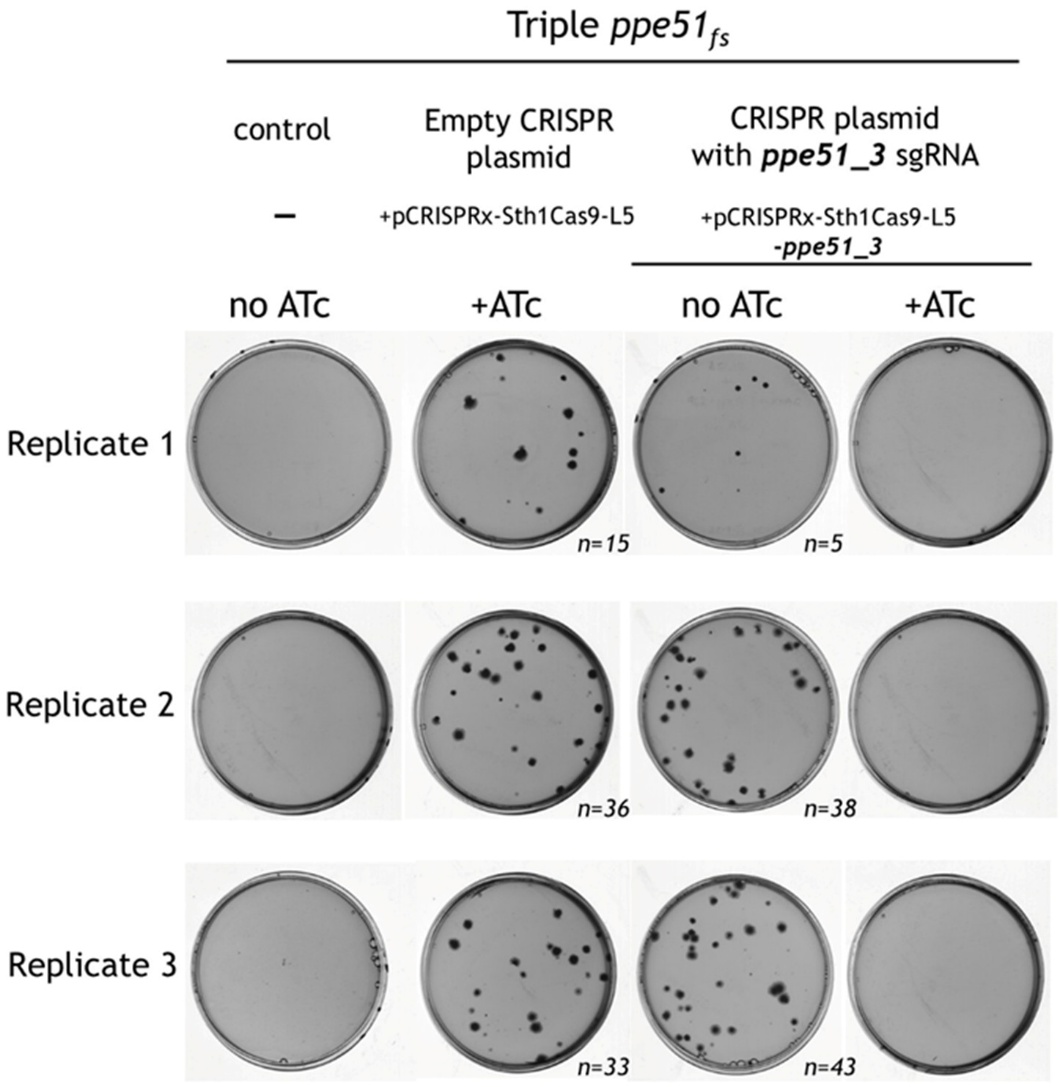


**Figure S1. Attempts to generate a quadruple *ppe51* frameshift mutant using CRISPR-Cas9.**
Triple *ppe51* frameshift mutant (*ppe51_fs_*, *ppe51_1_fs_*, *ppe51_2_fs_*) was transformed in three independent biological replicates with a CRISPR-Cas9 plasmid containing either a guide RNA targeting *ppe51_3* or no guide RNA. Transformants were only recovered when the CRISPR-Cas9 system targeting *ppe51_3* was not induced by anhydrotetracycline (+ATc), suggesting that successful targeting of *ppe51_3* prevents mutant recovery.


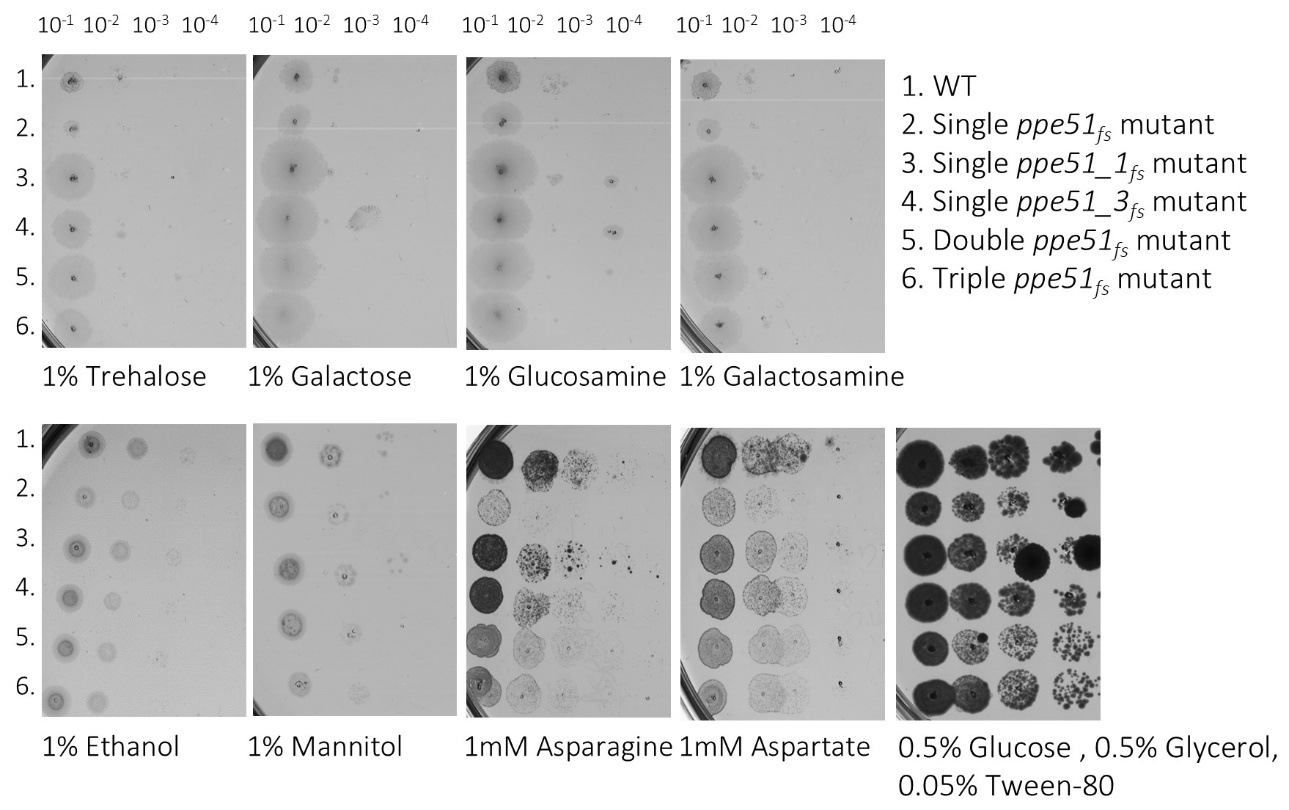


**Figure S2 Growth assays of *ppe51* frameshift mutants in different carbon and nitrogen sources.**

Single *ppe51* frameshift mutants, as well as double and triple mutants, were tested for their ability to grow on solid media when different carbon or nitrogen sources acted as sole nutrient sources. Defined medium containing 0.5% Glucose, 0.5% Glycerol, and 0.05% Tween-80 is used as a positive control.


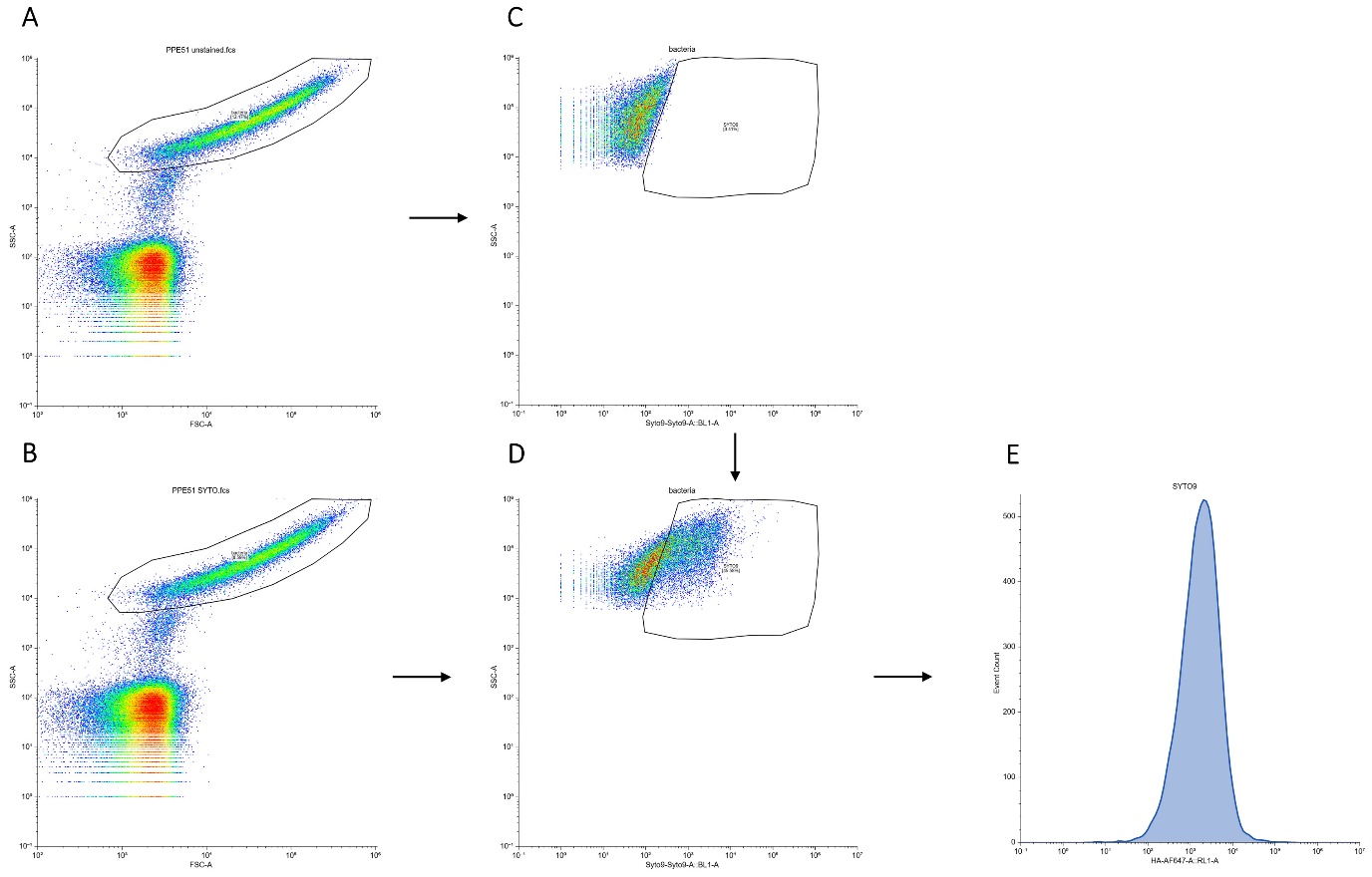


**Figure S3. Flow cytometry gating strategy used to identify bacterial populations expressing PPE51_tb_-HA.**

**A**. Unstained and **B**. Stained samples were first gated based on forward and side scatter (FSC/SSC) to select the bacteria. **C**. From this gate, SYTO9 fluorescence was plotted against side scatter for the unstained control. **D**. The same graph was generated for the stained sample. **E**. AF647 fluorescence of the SYTO9-positive population was measured to confirm fluorescence originating from bacteria expressing PPE51_tb_-HA.

*
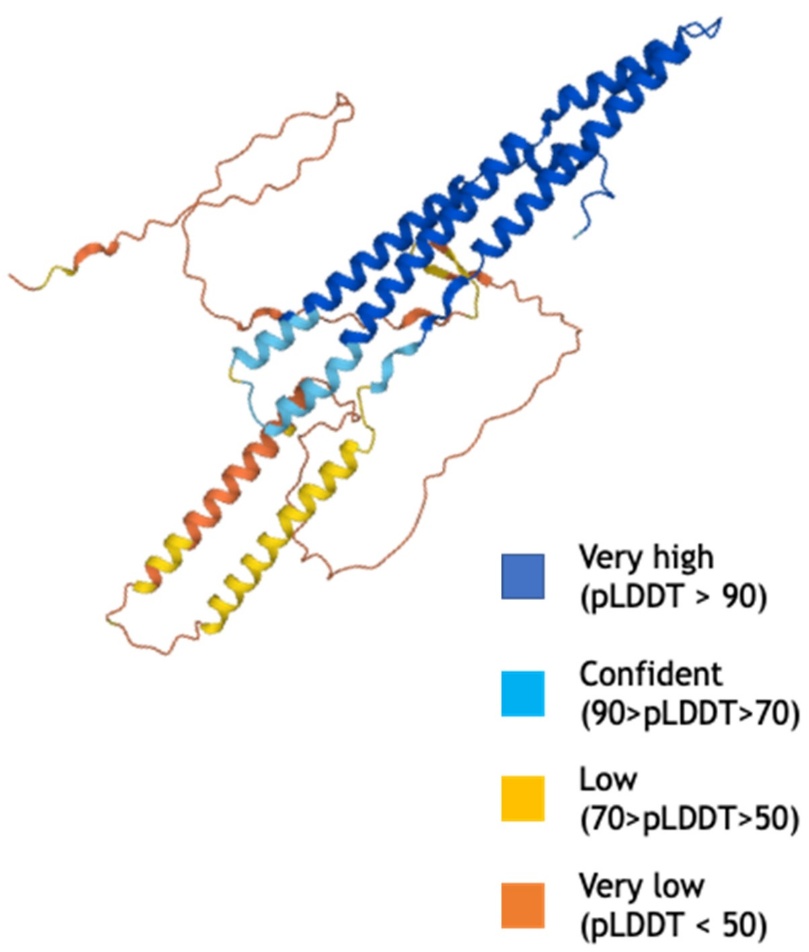
*

**Figure S4 PPE51 AlphaFold2 prediction of *Mycobacterium tuberculosis.***

**
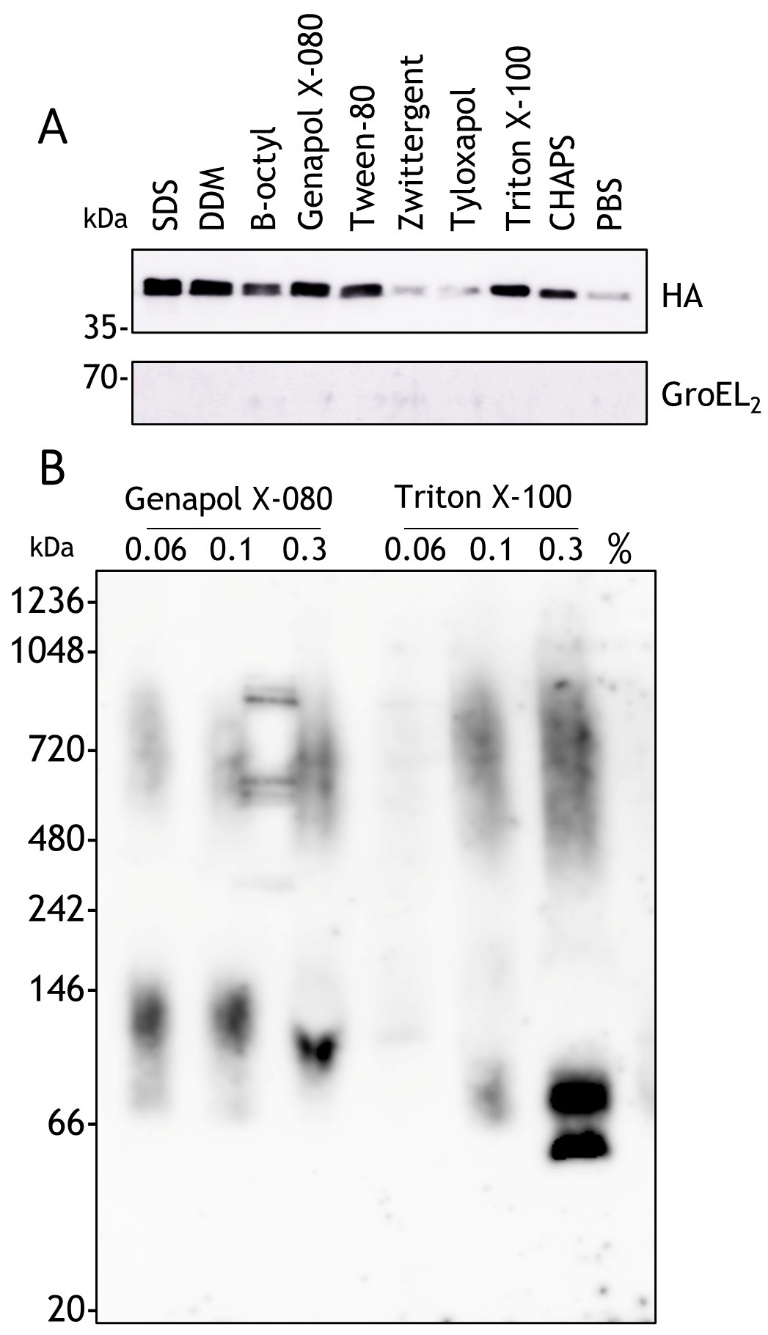
**

**Figure S5. Differential detergent extraction of PPE51_tb_-HA and analysis of complex formation in *M. marinum*.**

**A.** Screening of different detergents for PPE51_tb_-HA extraction in *M. marinum* WT (*pSMT3-pe19_tb_-ppe51_tb_-HA*). Intact bacteria were incubated with detergents at 1% concentration (SDS, DDM, β-octyl, Genapol X-080, Tween-80, Zwittergent, Tyloxapol, Triton X-100, CHAPS), and supernatants were collected to detect PPE51_tb_-HA by immunoblotting. SDS and PBS were included as extraction controls. A whole-cell lysate (WCL) sample was included as a reference. Detection of GroEL_2_ served as a lysis control. **B.** Blue Native PAGE (BN‑PAGE) analysis of PPE51tb‑HA complex formation at the bacterial surface. *ppe51* triple mutant cells overexpressing PPE51tb‑HA were incubated with Genapol X‑080 and Triton X‑100 at concentrations of 0.06%, 0.1%, and 0.3%. Surface-extracted PPE51tb‑HA was analyzed by BN‑PAGE followed by immunoblotting.

**
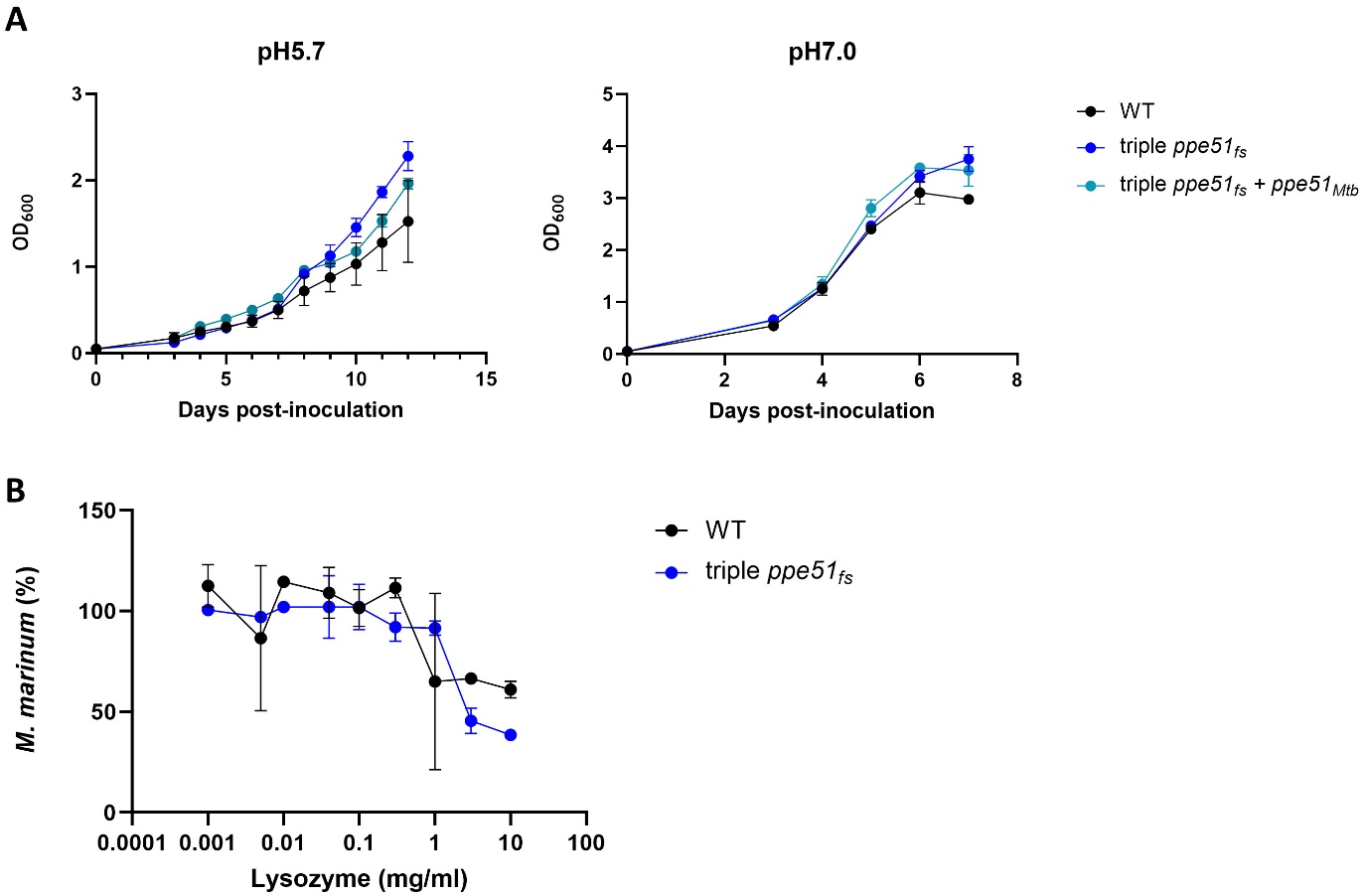
**

**Figure S6. Response of the triple *ppe51_fs_* mutant to host-relevant stress conditions.**

**A.** Growth curves of WT bacteria, the triple *ppe51_fs_* mutant, and the complementation strain (triple *ppe51_fs_* mutant overexpressing PPE51_tb_) in 7H9 medium supplemented with ADC and tyloxapol (0.02%) adjusted to pH 5.7 or pH 7.0. **B.** Relative growth of WT strain in the presence of lysozyme, assessed by resazurin reduction assay (REMA). Lysozyme was tested at concentrations ranging from 0.001 to 10 mg/mL.

**
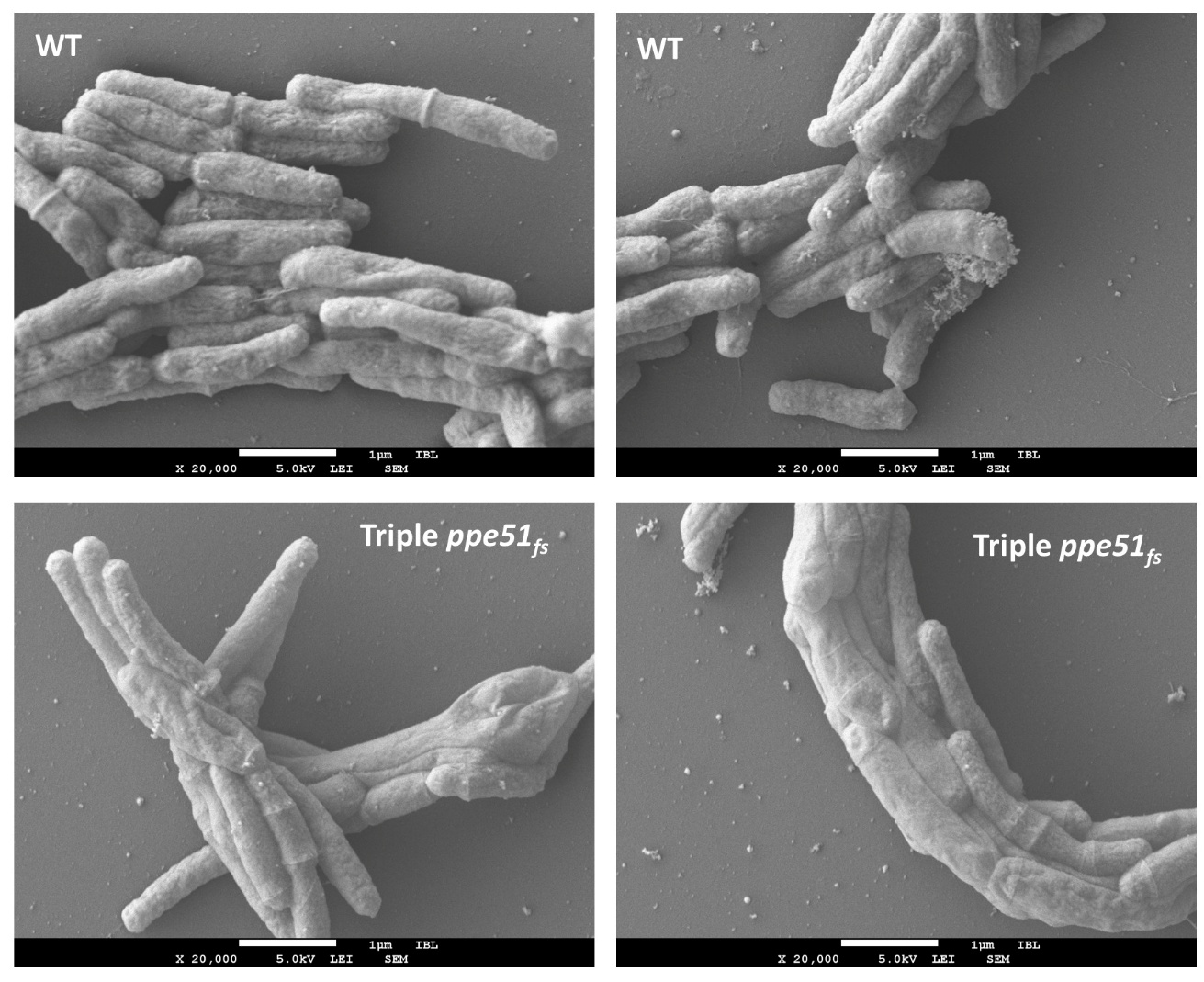
**

**Figure S7 Scanning Electron Microscopy (SEM) pictures.**

Wild type (WT) cells and triple ppe51_fs_ mutant cells at a magnification of 20,000 fold.

**Supplemental Tables:**

**Table S1:** Sequence alignment of the PPE51 protein from *M. tuberculosis* with all four PPE51 paralogues in *M. marinum.*

Rv3136|PPE51 MDFALLPPEVNSARMYTGPGAGSLLAAAGGWDSLAAELATTAEAYGSVLSGLAALHWRGP 60

MMAR_1513|PPE51 MDFSLLPPEVNSARMYTGPGSGSLLAAAASWDALAAELGTTAETCGSILTGLTDLSWRGP 60

MMAR_1514|PPE51_1 MNYAFLPPEVNSARMYSGPGSASLLAAAASWDMVAIELASAAEGYRSVISTLSGMYWWGP 60

MMAR_0191|PPE51_2 MDFAMLPPEVNSGLMYTGPGAGSLLAAANSWDELAIELGTTAQTYESVLSGLTSMYWAGP 60

MMAR_3465|PPE51_3 MNYAFLPPEVNSARMYSGPGPESLLAAAASWDMVAIELASTAEGYRSVISTLSGMYWWGP 60

*::::*******. **:*** ****** .** :* **.::*: *::: *: : * **

Rv3136|PPE51 AAESMAVTAAPYIGWLYTTAEKTQQTAIQARAAALAFEQAYAMTLPPPVVAANRIQLLAL 120

MMAR_1513|PPE51 ASESMAASAAPYVGWLYRTAEQTQQAAIQARTAALAFEQAYAMTVPPPLVAANRAQLLVL 120

MMAR_1514|PPE51_1 ASAAMLAATGPFIDWLEITGALASETANQAAAAAAAYEQAYAMTVPPIVVAANRALLVAL 120

MMAR_0191|PPE51_2 ASESMAAKAAPYVWWLHSTAEQTQQTATQCRAAAMAFEQAYAMTVPPPVILANRMQLMSL 120

MMAR_3465|PPE51_3 ASAAMLAATGPFIDWLEITGALASETANQATAAAAAYEQAYAMTVPPIVVAANRALLAAL 120

*: :* . :.*:: ** *. :.::* *. :** *:*******:** :: *** * *

Rv3136|PPE51 IATNFFGQNTAAIAATEAQYAEMWAQDAAAMYGYATASAAAALLTPFSPPRQTTNPAGLT 180

MMAR_1513|PPE51 IATNFFGQNTSAIAANEAQYAEMWAQDAAAMYGYATTSAAAGMLTPFSSPNQATSPAGLA 180

MMAR_1514|PPE51_1 VASNFFGQNTAAIEATEAEYAEMWAQDAGAMYDYAATSAMATALVPFSAPGQDTNPAGLA 180

MMAR_0191|PPE51_2 VATNFFGQNTAAIAALEAQYAEMWEQDAAAMYGYATSAAMAAQLVPFSSPQQTTNPAGLS 180

MMAR_3465|PPE51_3 IASNIFGQNTAAIEATEAEYAEMWAQDAGAMYDYAATSAMATALEPFPGPQQHTNPAGLP 180

:*:*:*****:** * **:***** ***.***.**:::* * * ** * * *.****

Rv3136|PPE51 AQAAAVSQ--------ATDPLSLLIETVTQALQA--LTIPSFIPEDFTFLDAIFAGYATV 230

MMAR_1513|PPE51 AQSTAVTQANATA--AATDPITQLVSSLTQSLGAI-QAIPSILPDDFTILDGVFSAYATV 237

MMAR_1514|PPE51_1 AQSAAVSQAAGNAPASAQSVWSQLTSLVPDALPSL-SNWPNILPDDFSILDGIFAMYATV 239

MMAR_0191|PPE51_2 AQSAAVNQADATA--AAANPLSDLIALVQKGLAALAGAFPNLLPDDFSILDGIFAGYATV 238

MMAR_3465|PPE51_3 AQGAAVSKAAASSEEGSQSLLSQLIALVPQALQVL-GIWP-ILPEEFTLLDAIFAMYATV 238

**.:**.: : . : * : ..* * ::*::*::**.:*: ****

Rv3136|PPE51 GVTQDVESFVAGTIGAESNLGLLNVGDENPAEVTPGDFGIGELVSATSPGGGVSASGAGG 290

MMAR_1513|PPE51 GVTQDIESFCAGVIGAENNLGLIGS-SENPAEVTPSDFGLGSLISSTAPASAASGGG--- 293

MMAR_1514|PPE51_1 GVTQDVESFCAGIIGAENNLGLLGAASENPAEVTPGALGLGAMFSSAERSAV-------- 291

MMAR_0191|PPE51_2 GVSQDCESIIAGIIGAESNLGMLGAASENPAELAPGDIGIGSVLSSAPSVGG-------- 290

MMAR_3465|PPE51_3 GMTADAVTIPVAIIAADNNLGLLGAASENPAELAPGALGVGAKLSSAEMGAA-------- 290

*:: * :: .. *.*:.***::. .*****::*. :*:* .*:: .

Rv3136|PPE51 AASVGNTVLASVGRANSIGQLSVPPSWAAPSTRPVSALSPAGLTTLPGTDVA-EHGMPGV 349

MMAR_1513|PPE51 ---LGSAVAASVGRSGSIGQLSVPPSWSAPSARMVSALSPSGLTTIPGTEEAAAAGSPGF 350

MMAR_0191|PPE51_2 AVSGPGAVLVSVGRAGSIGQLSVPASWAAPATRQVAALSSAGLTTIPGTEEA-AHGIPGM 349

MMAR_1514|PPE51_1 -GGATNAVTVSMSRAGSIGQLSVPPSWAAPSSGSASALSVSGLTTIPGTEVA-EQGAPGM 349

MMAR_3465|PPE51_3 -RGAANAVTVSMSRAGSIGQLSVPPSWAAPSSGSASALSVSGLTTIPGTEVA-EQGAPGV 348

.:* .*:.*:.******** **:**:: .:*** :****:***: * * **.

Rv3136|PPE51 PGVP-VAAGRASGVLPRYGVRLTVMAHPPAAG 380

MMAR_1513|PPE51 PGMVAPPATRASGVLPRYGVRLTVMAHPPAAG 382

MMAR_1514|PPE51_1 PGMPVGTGKRATSVIPRYGVRLTVMARPPAAG 381

MMAR_3465|PPE51_3 PGMPVGTGKRATSVIPRYGVRLTVMARPPAAG 380

MMAR_0191|PPE51_2 PGMPVGSSSRASSVVPRYGVRLTVMAHPPAAG 381

**: . **:.*:***********:*****

**Table S2.** Strains used in this study.

| **Strains** | **Referred to as** | **Characteristics** | **References** |
| --- | --- | --- | --- |
| *M. tuberculosis* H37Rv mc^2^6030 | *M. tuberculosis* WT | Δ*RD1* Δ*panCD* | ^1^ |
| *M. marinum* WT | WT | wild type M^USA^ | ^2^ |
| *M. marinum ΔeccC_b1_* | Δ*eccC1* | *mmar_5444.eccCb1_fs_* | ^3^ |
| *M. marinum ΔeccC_4_* | Δ*eccC4* | *mmar_1102.eccC_4_*::*res* | ^4^ |
| *M. marinum ΔeccC_5_ +* pSMT3::*mspA* | Δ*eccC5* | *mmar_2665. eccC5*::*loxP*, pSMT3::*mspA* | ^5^ |
| *M. marinum ppe51_fs_* | *ppe51_fs_* | *mmar_1513:179-180del, L5::*pCRISPRx-Sth1Cas9 | This study |
| *M. marinum ppe51_fs__ppe51_1_fs_* | double *ppe51_fs_* | *M. marinum ppe51_fs_* derivative *, mmar_1514: 69‑72del, L5::* pCRISPRx-Sth1Cas9 | This study |
| *M. marinum ppe51_fs__ppe51_1_fs__ ppe51_2_fs_* | triple *ppe51_fs_* | *M. marinum ppe51_fs__ppe51_1_fs_ derivative, mmar_0191: 154insC,  L5::* pCRISPRx-Sth1Cas9 | This study |
| *M. marinum ppe51_fs__ppe51_1_fs__ ppe51_2_fs__ ppe51_3_fs_* +pSMT3::*pe19*-*ppe51tb* | - | *M. marinum ppe51_fs__ppe51_1_fs__ ppe51_2_fs_ derivative, mmar_3465*:506‑512del,  *L5::* pCRISPRx-Sth1Cas9, pSMT3::*pe19*-*ppe51*_1mmar | This study |
| *M. marinum ppe51_fs__ppe51_1_fs__ ppe51_2_fs__ ppe51_3_fs_* +pSMT3::*pe19*-*ppe51mmar* | - | *M. marinum ppe51_fs__ppe51_1_fs__ ppe51_2_fs_ derivative, mmar_3465*:506‑512del,  *L5::* pCRISPRx-Sth1Cas9, pSMT3::*mmar_2670-mmar_1514* | This study |
| *M. marinum* WT *+* pSMT3::*mspA* | WT + MspA | wild type M^USA^ | ^6^ |
| *M. marinum ppsE_fs_* | *ppsE_fs_* | *mmar_1772*:1522insA*, L5::* pCRISPRx-Sth1Cas9 | ^7^ |

**Table S3.** Oligonucleotides used to construct frameshift mutants in this study.

| **Oligonucleotide** | **Sequence** | **PAM sequence** | **Forward primer 5'-3' (BsmI NEB)** | **Reverse primer 5'-3' (BsmI NEB)** |
| --- | --- | --- | --- | --- |
| *ppe51_mar_*-sgRNA | TGAGTTGGCGTGGCCCGGCA | TCGGAAT | GGGATGAGTTGGCGTGGCCCGGCA | AAACTGCCGGGCCACGCCAACTC |
| *ppe51_1*-sgRNA | CCGCGGCGGCCAGTAGCGAC | GCAGAAC | GGGACCGCGGCGGCCAGTAGCGAC | AAACGTCGCTACTGGCCGCCGCGG |
| *ppe51_2*-sgRNA | ATCGGTGCTATCGGGCCTGA | CGAGCAT | GGGAATCGGTGCTATCGGGCCTGA | AAACTCAGGCCCGATAGCACCGAT |
| *ppe51_3*-sgRNA | TTGGTGTGCTGTTGTGGCCC | GGGGAAC | GGGATTGGTGTGCTGTTGTGGCCC | AAACGGGCCACAACAGCACACCAA |

**Table S4.** Plasmids used in this study

| **Name** | **Features** | **References** |
| --- | --- | --- |
| pSMT3-mspA | p_hsp60_-*mspA*; *pMP1* origin; *pAL5000* origin; *hyg* | ^8^ |
| pSMT3-PE19_tb_ | p_hsp60_-*rv1791*; *pMP1* origin; *pAL5000* origin; *hyg* | This study |
| pSMT3-PE19_tb_-PPE51_tb_ | p_hsp60_-*rv1791-rv3136*; *pMP1* origin; *pAL5000* origin; *hyg* | This study |
| pSMT3-PE19_mmar_-PPE51_mmar_ | p_hsp60_- *mmar_2670-mmar_1514*; *pMP1* origin; *pAL5000* origin; *hyg* | This study |
| pSMT3- PE19_tb_-PPE51_tb_-HA | p_hsp60_-*rv1791-rv3136*-HA; *pMP1* origin; *pAL5000* origin; *hyg* | This study |
| pCRISPRx-Sth1Cas9-L5 | *Sth1Cas9*; KanR versions; L5 integrase | ^9^ |
| pCRISPRx-Sth1Cas9-L5-Hyg | *Sth1Cas9*; HygR versions; L5 integrase | ^9^ |
| pCRISPRx-Sth1Cas9-L5-Hyg-*ppe51_mar_* | *Sth1Cas9;* HygR; L5 integrase; *ppe51_mar_*-sgRNA | This study |
| pCRISPRx-Sth1Cas9-L5-*ppe51_1* | *Sth1Cas9;* KanR; L5 integrase; *ppe51_1*-sgRNA | This study |
| pCRISPRx-Sth1Cas9-L5-Hyg-*ppe51_2* | *Sth1Cas9;* HygR; L5 integrase; *ppe51_2*-sgRNA | This study |
| pCRISPRx-Sth1Cas9-L5-*ppe51_3* | *Sth1Cas9;* KanR; L5 integrase; *ppe51_3*-sgRNA | This study |

**Table S5.** Oligonucleotides used in this study

| **Oligonucleotide** | **Sequence 5’-3’** |
| --- | --- |
| V01 | GGAGGAATCACGCTAGCATGTCGTTCGTGACCACACAGCCGG |
| V02 | TAGATATCCATGGATCCTCAGCCGGCAGCGGCTGCGT |
| V03 | GGAGGAATCACGCTAGCATGTCGTTTGTGACCACACAGCCGGAG |
| V04 | TAGATATCCATGGATCCTCAGCCGGCAGCGGCCGCGT |
| V05 | CTGCCGGCTGAGGATCCATGGATTTCGCACTGTTACCACCGG |
| V06 | GTGGCGGCCGCTCTAGATTACCCTGCCGCGGGTGGGT |
| V07 | CTGCCGGCTGAGGATCCATGAACTACGCATTCTTGCCGCCCGAAG |
| V08 | GTGGCGGCCGCTCTAGATCAGCCGGCCGCGGGCGGGC |
| V09 | GTCCGGCACGTCGTACGGGTAACTAGTCCCTGCCGCGGGTGGGTGGG |
| V10 | GTGGCGGCCGCTCTAGATCACGCGTAGTCCGGCACGTCGTACGGGT |

1 Sambandamurthy, V. K. *et al.* Mycobacterium tuberculosis ΔRD1 ΔpanCD: A safe and limited replicating mutant strain that protects immunocompetent and immunocompromised mice against experimental tuberculosis. *Vaccine* **24**, 6309-6320 (2006). <https://doi.org/10.1016/J.VACCINE.2006.05.097>

2 Abdallah, A. M. *et al.* A specific secretion system mediates PPE41 transport in pathogenic mycobacteria. *Molecular Microbiology* **62**, 667-679 (2006). <https://doi.org/10.1111/j.1365-2958.2006.05409.x>

3 Pym, A. S. *et al.* Recombinant BCG exporting ESAT-6 confers enhanced protection against tuberculosis. *Nature Medicine* **9**, 533-539 (2003). <https://doi.org/10.1038/nm859>

4 Izquierdo Lafuente, B., Ummels, R., Kuijl, C., Bitter, W. & Speer, A. Mycobacterium tuberculosis Toxin CpnT Is an ESX-5 Substrate and Requires Three Type VII Secretion Systems for Intracellular Secretion. *mBio* **12**, 1-16 (2021). <https://doi.org/10.1128/MBIO.02983-20>

5 Damen, M. P. M. *et al.* Modification of a pe/ppe substrate pair reroutes an esx substrate pair from the mycobacterial esx-1 type VII secretion system to the esx-5 system. *Journal of Biological Chemistry* **295**, 5960-5969 (2020). <https://doi.org/10.1074/jbc.RA119.011682>

6 Ates, L. S. *et al.* Essential Role of the ESX-5 Secretion System in Outer Membrane Permeability of Pathogenic Mycobacteria. *PLoS Genetics* **11**, e1005190-e1005190 (2015). <https://doi.org/10.1371/JOURNAL.PGEN.1005190>

7 Izquierdo Lafuente, B. *et al.* Vitamin B 12 uptake across the mycobacterial outer membrane is influenced by membrane permeability in Mycobacterium marinum. *Microbiology Spectrum* **12** (2024). <https://doi.org/10.1128/SPECTRUM.03168-23>

8 Ates, L. S. New insights into the mycobacterial PE and PPE proteins provide a framework for future research. *Molecular microbiology* **113**, 4-21 (2020). <https://doi.org/10.1111/MMI.14409>

9 Meijers, A. S. *et al.* Efficient genome editing in pathogenic mycobacteria using Streptococcus thermophilus CRISPR1-Cas9. *Tuberculosis* **124**, 101983-101983 (2020). <https://doi.org/10.1016/j.tube.2020.101983>

**Supplementary References:**
